# Tolerant and Susceptible Sesame Genotypes Reveal Waterlogging Stress Response Patterns

**DOI:** 10.1371/journal.pone.0149912

**Published:** 2016-03-02

**Authors:** Linhai Wang, Donghua Li, Yanxin Zhang, Yuan Gao, Jingyin Yu, Xin Wei, Xiurong Zhang

**Affiliations:** Oil Crops Research Institute of the Chinese Academy of Agricultural Sciences, Key Laboratory of Biology and Genetic Improvement of Oil Crops of the Ministry of Agriculture, Wuhan, China; National Key Laboratory of Crop Genetic Improvement, CHINA

## Abstract

Waterlogging is a common adverse environmental condition that limits plant growth. Sesame (*Sesamum indicum*) is considered a drought-tolerant oil crop but is typically susceptible to harmful effects from waterlogging. The present study used comparative analysis to explore the waterlogging stress response associated with two sesame genotypes. The RNA-seq dataset generated during a time course of 0, 3, 9 and 15 h of waterlogging as well as 20 h post-drainage indicated that stress gradually suppressed the expression of sesame genes, with 9 h as the critical time point for the response of sesame to waterlogging stress. Of the 19,316 genes expressed during waterlogging, 72.1% were affected significantly. Sesame of both tolerant and susceptible genotypes showed decreased numbers of upregulated differentially expressed genes (DEGs) but increased numbers of downregulated DEGs at the onset of waterlogging. However, the tolerant-genotype sesame exhibited 25.5% more upregulated DEGs and 29.7% fewer downregulated DEGs than those of the susceptible-genotype strain between 3 and 15 h. The results indicated that the tolerant sesame displayed a more positive gene response to waterlogging. A total of 1,379 genes were significantly induced and commonly expressed in sesame under waterlogging conditions from 3 to 15 h regardless of tolerance level; of these genes, 98 are known homologous stress responsive genes, while the remaining 1,281 are newly reported here. This gene set may represent the core genes that function in response to waterlogging, including those related mainly to energy metabolism and phenylpropanoid biosynthesis. Furthermore, a set of 3,016 genes functioning in energy supply and cell repair or formation was activated in sesame recovery from waterlogging stress. A comparative analysis between sesame of the tolerant and susceptible genotypes revealed 66 genes that may be candidates for improving sesame tolerance to waterlogging. This study provided a comprehensive picture of the sesame gene expression pattern in response to waterlogging stress. These results will help dissect the mechanism of the sesame response to waterlogging and identify candidate genes to improve its tolerance.

## Introduction

Crops have developed a variety of adaptations to natural and agronomic habitats that have contributed to their survival and regeneration over the course of evolution. However, abnormal or altered climates tend to overwhelm the endurance capacity of a crop, imposing severe negative influences on the productivity of arable farmland as the vast majority of crops are ill-suited to grow under stress conditions. Waterlogging is a common adverse environmental condition that limits plant growth. Waterlogging reduces gas exchange between plant tissues and the atmosphere, resulting in an imbalance between slow diffusion and rapid consumption of oxygen in the rhizosphere that drastically reduces the oxygen supply and induces anoxia in plants [1].

Sesame, a crop with high oil content, has the potential capacity to combat nutritional deficiencies in developing regions and countries. Most current cultivars contain 50–60% oil and 18–24% protein in their seeds [2, 3] (http://ndb.nal.usda.gov/). In particular, greater than 80% of its oil is in the form of unsaturated fatty acids, which are more beneficial for human health than are saturated fatty acids. In addition, the antioxidant properties of sesame lignans, primarily sesamin and sesamolin, are used for therapeutic and cosmetic applications [4, 5]. Sesame is typically considered drought-tolerant but susceptible to waterlogging, a property that can be ascribed to its suspected origin in Africa or India and its subsequent dispersal to tropical or semitropical regions [3, 6]. According to the Food and Agricultural Organization, the average sesame yield was alarmingly low at only 617 kg/ha worldwide in 2011 and ranked second to last among 22 oil crops between 2007 and 2011 (http://faostat.fao.org). This low yield may be attributed to several reasons, but waterlogging is a primary factor that has a severe effect in countries such as China and Korea due to changing climate.

To understand the effects of abiotic stress in an effort to maintain a stable food supply, a number of studies have investigated the responses of model plants and crops to stresses [7]. These studies have revealed that plant responses to different stresses are coordinated by complex and often interconnected signaling pathways that regulate numerous metabolic networks [8, 9]. At the protein level, low oxygen selectively induces the synthesis of anaerobic proteins, especially enzymes involved in sugar metabolism, glycolysis and fermentation [10, 11]. The vast majority of these proteins have been investigated in waterlogging-susceptible or -tolerant strains of *Arabidopsis* or rice [8, 12, 13]. Despite an increased understanding of the adaptive mechanisms and molecular regulation at play in other crops, the mechanisms underlying the sesame response to waterlogging need further elucidation [14, 15].

With the recent completion of the *de novo* assembly of the sesame genome [16, 17], it is now possible to profile gene expression in response to waterlogging by sequencing [15, 18]. Thus, the present study explored the molecular mechanisms of the sesame response to waterlogging stress by performing a comparative RNA-seq-based analysis between waterlogging-tolerant and -susceptible genotypes.

## Materials and Methods

### Materials

The waterlogging-tolerant cultivar Zhongzhi No. 13 (WT) and the waterlogging-susceptible strain ZZM0563 (WS) were used in this study. The two plants were potted under the same growth and experimental conditions and treated with waterlogging simultaneously for 15 h during the flowering stage. For the sesame of both genotypes, a total of 10 samples were collected at equivalent stages: 0, 3, 9, and 15 h under waterlogging stress as well as 20 h post-drainage (S1 Table). For each sample, the root tips of five plants were obtained and mixed. All samples were subjected to RNA-seq analysis.

### RNA extraction and library preparation

RNA extraction and sequencing were performed according to the procedure described by Wei *et al*. [19]. Total RNA was extracted from 10 samples using TRIzol reagent (Invitrogen), and its concentration and quality were tested using an ultraviolet spectrophotometer and denaturing agarose gels (1.0%). Next, the RNA was treated with DNase I and magnetic oligo (dT) beads. Divalent cations and heat were used to fragment the purified poly-(A) mRNA before library construction. The RNA fragments were transcribed into double-stranded cDNA, which were subjected to end repair and index adapter ligation using T4 DNA polymerase. The adapter-ligated cDNA fragments of the desired size range (200 ± 25 bp) were excised from an agarose gel. The cDNA fragments were then enriched selectively via polymerase chain reaction (PCR).

### Data generation and quality assessment

The 10 cDNA libraries generated from RNA samples isolated from the roots of WT and WS at 0, 3, 9 and 15 h during stress and 20 h post stress (P20h) were paired-end sequenced using the Illumina platform. We used FastQC (http://www.bioinformatics.babraham.ac.uk/projects/fastqc/) to determine the base quality of the RNA-seq reads and removed the paired-end reads containing more than 5% ambiguous residues (Ns) and those containing > 10% bases with a Phred quality score < 20. The remaining reads were referred to as “clean reads”[20]. After cleaning and quality assessment, approximately 25.6–26.8 million high-quality reads of 90-bp lengths remained in each sample (S1 Fig and S2 Table). The high-quality reads were mapped to a reference genome using SOAPaligner/SOAP2 [16, 21, 22], allowing no more than one mismatch in the alignment. Approximately 65.1–81.4% of the clean reads were uniquely mapped to the reference genome [16], with 50.3–64.2% of them uniquely mapped to genic regions. Most gene models were covered by reads over 80% (S2 Fig), and the reads were evenly distributed on these genes (S3 Fig).

### Statistical analysis of gene expression

Gene expression levels were calculated based on the number of unique matched reads to the sesame genome [16] and were normalized to reads per kilobase of transcript per million mapped reads (RPKM) using Cufflinks 2.0 software [23]. Differentially expressed genes (DEGs) were identified as described by Chen et al. [24] and Wang et al. [15] based on a Poisson distribution [25]. In addition to *P*-values, false discovery rates (FDR) were used to determine the threshold *P*-value in multiple tests. FDR ≤ 0.001 and the absolute value of log_2_Ratio ≥ 1 were used as thresholds to determine the significance of the DEGs [26].

### Gene annotation and enrichment analysis

Target genes were annotated by gene ontology (GO; http://www.geneontology.org) and metabolic pathway. GO is an international standardized gene function classification system that offers a dynamically updated, controlled vocabulary and a strictly defined conceptual framework for the comprehensive description of gene properties and products in any organism. GO term and metabolic pathway enrichment analyses were performed according to Wang et al. [15]. GO terms were summarized using the online tool GOSlimAuto [27].

### Homologous stress-responsive genes and hormone-related genes

The 3,150 biotic and abiotic stress-responsive genes of *Arabidopsis thaliana* were downloaded from the Stress Responsive Transcription Factor Database (STIFDB V2.0) [28]. The 1,318 *Arabidopsis* hormone-related genes were downloaded from *Arabidopsis* Hormone Database 2.0 (AHD2.0) [29]. Then, their homologous genes in sesame were predicted using the Reciprocal Best Blast Hit (RBH) method [30, 31].

### Real-time quantitative PCR (qRT-PCR)

RT-PCR analyses of target gene expression were performed according to Wang et al. [15] using the iQ^™^5 Real-Time PCR Detection System (Bio-Rad, San Diego, CA, USA). Samples were run in triplicate on the same plate with a negative control that lacked cDNA. The positive control for each sample was the sesame gene ubiquitin-conjugating enzyme 9 (UBC9). The PCR efficiency was determined by a series of 10-fold dilutions of cDNA. The calculated efficiency of all primers was 0.9 to 1.0. The relative expression levels of the genes were calculated using the 2^-ΔΔCT^ method [32].

## Results and Discussion

### WT and WS showed similar expressed gene numbers under waterlogging stress

**Fig 1:**
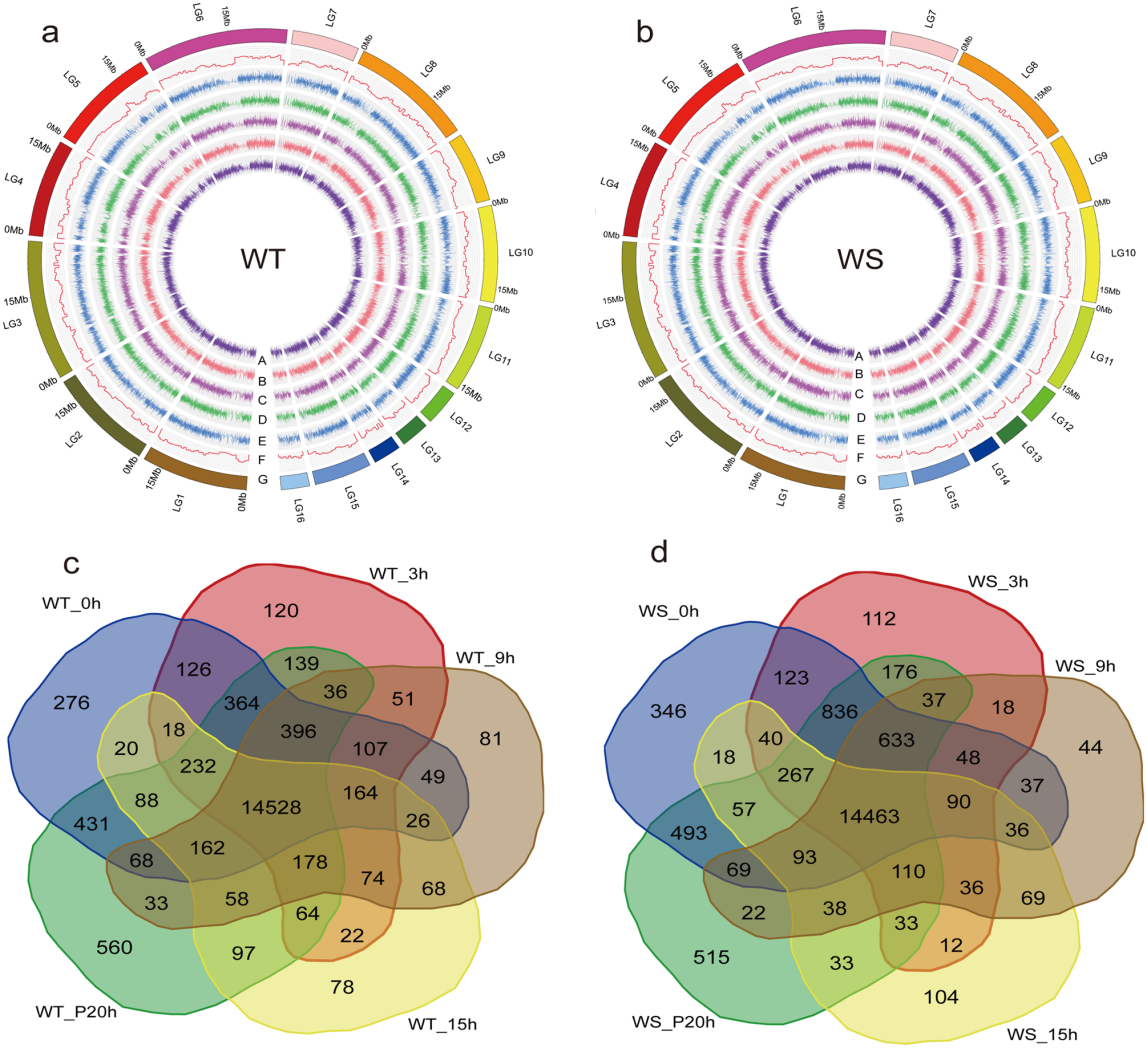
An overview of gene expression in WT and WS plants. (a) and (b) indicate the gene expression levels (RPKM ≥ 1, RPKM: reads per kilobase of transcript per million mapped reads) in WT and WS plants at each time point, respectively. (A), (B), (C), (D), and (E) correspond to the samples at 0, 3, 9, and 15 h during waterlogging and 20 h post drainage, respectively. (F) represents the gene density (mRNA, 500-kb window) in the sesame linkage groups, and (G) represents the 16 assembled pseudomolecules of sesame. (c) and (d) indicate the shared and uniquely expressed gene numbers during a time-point assay of waterlogging. WT: waterlogging-tolerant sesame genotype, WS: waterlogging-susceptible sesame genotype.

After mapping the reads to the reference sequence [16], gene expression levels were calculated as RPKMs. A range of 15,499 to 17,875 genes was expressed at each time point, with RPKMs ranging from 1 to 23,367 and averaging 48.6 (Fig 1a and 1b). Comparative numbers of genes were expressed in the sesame of both genotypes (18,714 in WT and 19,008 in WS)) (Fig 1c and 1d), resulting in a total of 19,316 genes expressed. Of these genes, 14,528 and 14,463 were expressed commonly at each time point in WT and WS plants, respectively, with 13,959 shared by the two genotypes (Figs 1b, 1d and S4), accounting for approximately 51.4% of sesame genes. The activation of these genes before and during waterlogging and post-drainage suggests their importance in survival of sesame. GO enrichment of the common gene set indicated that most genes were involved in metabolic processes and were strongly associated with initiating functions, such as protein binding, small molecule binding, nucleotide binding and nucleic acid binding (S5 Fig). In addition to the genes commonly expressed between WT and WS, there were 569 genes uniquely expressed in WT and 504 in WS (RPKM ≥ 1) (S4 Fig). The 569 genes in WT were enriched in the molecular function term imidazoleglycerol-phosphate dehydratase (IGPD) activity, but no GO terms were enriched among the 504 genes in WS. IGPD catalyzes the sixth step in the histidine biosynthesis pathway, an essential process in plants and microorganisms [33]. We speculated that IGPD genes might help WT endure waterlogging stress.

### Gradual suppression of expressed genes by waterlogging

**Fig 2:**
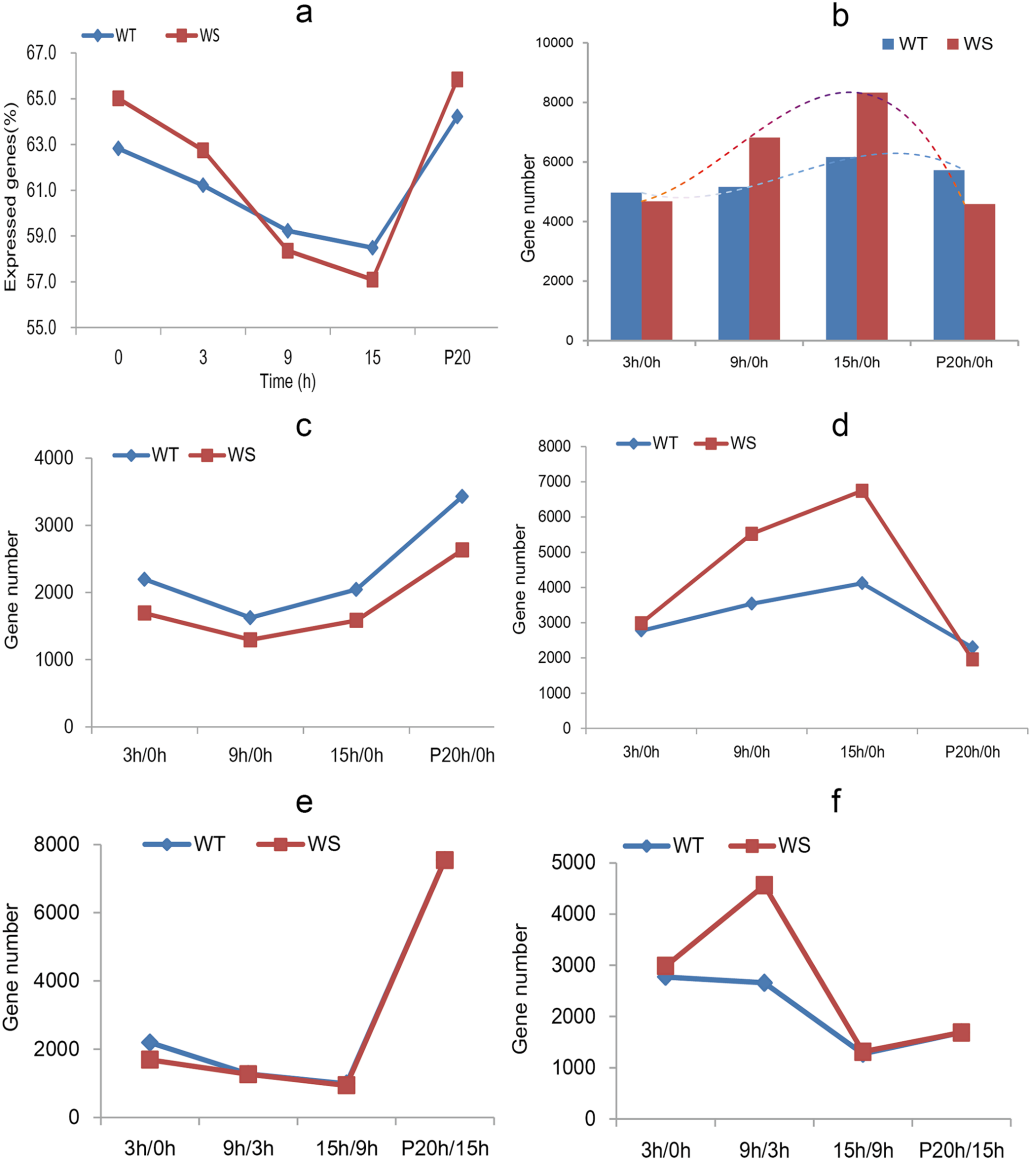
Changes in gene expression in WT and WS plants. (a) Percentages of the expressed genes during waterlogging from 0 to 15 h as well as 20 h post drainage. (b) The number of genes significantly affected by the stress. (c) Upregulated genes using 0 h as a control. (d) Downregulated genes using 0 h as a control. (e) Upregulated genes using the former sampling point as a control, illustrating the dynamic changes in differentially expressed genes (DEGs) in sesame upon waterlogging exposure. (f) Downregulated genes using the former sampling point as a control.

The statistical graph showed that the global transcriptional responses in root tissue were suppressed gradually by waterlogging, with the rate of expressed genes decreasing from 62.8 to 58.5% in WT, and from 65.0 to 57% in WS in the time-course assay under stress, from 3 to 9 h of waterlogging. When the water was removed (P20), many genes in the sesame of both genotypes resumed expression, but the numbers of active genes were lower in the tolerant plants compared with the susceptible plants (Fig 2a and S6 Fig).

Next, we determined how many of these genes were affected significantly by stress. Using the 0 h time point as a control, the DEGs at each time point were identified for the two genotypes. Of the 19,316 expressed genes in sesame under waterlogging stress, 72.1% were differentially expressed in the samples during waterlogging stress or post-drainage in both WT and WS, compared with 4–15% of the assayed genes in *Arabidopsis* [34], rape [18], cotton [35] and poplar [36]. This result suggested that most of these genes are affected by low rhizosphere oxygen or are involved in recovery. The greater preponderance of affected genes in sesame underscores its sensitivity to waterlogging. However, the tolerant strain exhibited a smoother trend in fluctuation than did the susceptible plants, suggesting its enhanced ability to stabilize metabolism to combat abiotic stress (Fig 2a and 2b).

For the remaining 5,397 genes that were not significantly affected by waterlogging stress, enrichment analysis revealed that most were associated with a few GO terms (P < 0.01) (S3 Table). Using GO-SLIM, these enriched insensitive genes were further categorized into fundamental biological processes, including cellular metabolic, primary metabolic, and biosynthetic and transport processes, together with binding functions, catalytic activity, oxidoreductase activity, kinase activity and transferase activity (S7 Fig). Thus, these insensitive genes may be primarily housekeeping genes.

### Sesame endurance to waterlogging showed a critical time point of 9 h

Over the time course of waterlogging stress, both WT and WS showed gradually increasing DEG numbers from 3 to 15 h (using the 0 h samples as controls), which decreased after the stress was removed. In comparison with the control (0 h), the number of upregulated DEGs decreased from 0 to 9 h in both genotypes and then increased, with 25.5 to 29.7% more upregulated DEGs in WT than WS over the treatment duration. However, the downregulated DEGs exhibited a different pattern, in that WT expressed 36.0% fewer downregulated DEGs than those of WS at 9 h and 15 h (Fig 2c and 2d), with no significant differences noted at the onset of waterlogging or post-drainage. By comparing each sample (except for 0 h) against those collected at the previous time-points, the immediate changes in DEG numbers in both WT and WS were examined. The two genotypes had similar numbers of upregulated genes at each time point but exhibited differences in downregulated gene numbers, in that more genes were downregulated in WS than WT at 9 h. These results demonstrated that 9 h was a critical time point for endurance of waterlogging stress in sesame (Fig 2e and 2f).

### The core gene set in sesame responsible for the response to waterlogging stress

**Fig 3:**
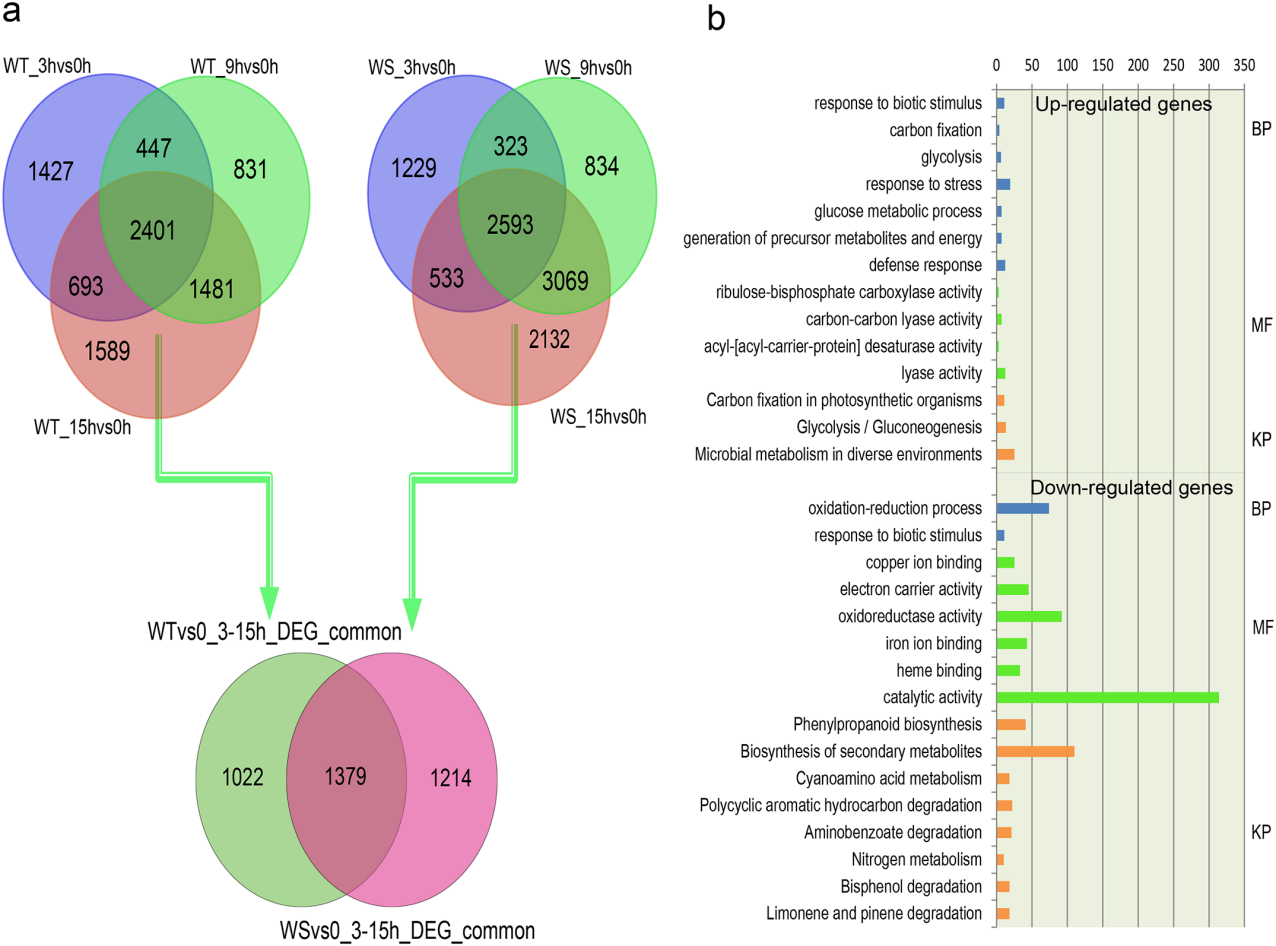
The core gene set responsible for waterlogging stress in sesame. (a) Venn diagrams of the special and unique DEGs at time points between 3 and 15 h under waterlogging conditions. (b) Enriched gene ontology (GO) and Kyoto Encyclopedia of Genes and Genomes (KEGG) pathway categories among the upregulated (up channel) and downregulated (down channel) genes in the core set.

WT and WS showed different DEG numbers over the course of waterlogging, using 0 h as control; 2,401 genes were commonly and differentially expressed in the tolerant strain from 3 to 15 h and 2,593 in the susceptible plant. Of these DEGs, 1,379 were shared by the two genotypes (Fig 3a and S4 Table). The shared genes may represent a core gene set in sesame that function in response to waterlogging stress, despite tolerance level. These 1,379 genes were further clustered into 18 groups, and various expression patterns of the genes in each group were observed (S8 Fig). Of these genes, 326 were upregulated, representing positive responses to waterlogging stress, whereas 839 were downregulated in both WT and WS strains.

Anoxic or hypoxic conditions of the rhizosphere affect the plant’s maintenance of numerous pathways [37]. To generate energy under conditions of low O_2_ availability, plants must regulate their molecular pathways to survive stress. Functional annotation of the 326 upregulated genes in response to stress revealed that sesame enlists the molecular functions of ribulose-bisphosphate carboxylase activity, carbon-carbon lyase activity, acyl-[acyl-carrier-protein] desaturase activity and lyase activity by activating various biological processes, such as those involved in the response to biotic stimuli (GO:0009607), response to stress (GO:0006950), defense response (GO:0006952), carbon fixation (GO:0015977), glycolysis (GO:0006096), glucose metabolism (GO:0006006) and generation of precursor metabolites and energy (GO:0006091).

Energy metabolism plays crucial roles in plant physiological adaptation to hypoxia [10]. Many studies have demonstrated that plants switch from respiration to fermentation as a rescue mechanism that regenerates NAD+ to maintain glycolysis and produce ATP under anaerobic conditions [11, 35, 36]. A high rate of fermentation increases the demand for carbohydrates. Kyoto Encyclopedia of Genes and Genomes (KEGG) analysis showed that carbon fixation in photosynthetic organisms, as well as glycolysis and gluconeogenesis, were enriched pathways among the upregulated genes (Figs 3b, S9 and S10). Carbon fixation is a process used by plants to generate energy under abiotic stress [38, 39] and occurs during the second half of the photosynthetic process of converting light to energy. The upregulation of genes related to carbon fixation and glycolysis suggests that sesame will invoke or enhance energy generation to maintain growth during long periods of root hypoxia [36].

In addition to upregulating genes involved in carbon fixation and fermentative metabolism [34], an alternative abiotic stress survival mechanism relies on decreased energy consumption. Protein degradation plays a negative regulatory role in response to hypoxia [40], resulting in large-scale energy savings under hypoxic conditions [41]. We found that waterlogging stress in sesame resulted not only in the downregulation of genes enriched in nitrogen metabolism and cyanoamino acid metabolism, but also in secondary metabolite and phenylpropanoid biosynthesis (Fig 3c). Coupled with these processes, oxidation-reduction processes usually slow down to reduce the release of biological energy [42, 43]. Phenylpropanoid biosynthesis is regulated by biotic and abiotic stimuli [44–46], and the phenylpropanoid-based polymers such as lignin, suberin and tannin contribute substantially to the stability and robustness of plants in the face of mechanical or environmental damage. Some of the homologous genes (SIN_1026962, SIN_1023248, SIN_1022230, SIN_1013457, SIN_1012086, SIN_1009800, SIN_1007000) involved in phenylpropanoid biosynthesis had been reported function in response to biotic or abiotic stress in *Arabidopsis* [47, 48]. Their downregulation in expression suggested the harmful effect of waterlogging. In plants, biotic and abiotic stressors tend to intersect. The presence of abiotic stress can result in the reduced enhancement of its susceptibility to a biotic pest or pathogen, and vice versa [49]. For example, a change in temperature or humidity may induce the expression of defense genes in response to certain pathogens. These results indicate that energy metabolism and phenylpropanoid biosynthesis play crucial roles in sesame endurance against waterlogging stress.

### The genes involved in recovery from waterlogging

After drainage, some sesame genes may be selectively employed in the recovery and reconstruction of metabolic processes. We observed that the number of DEGs differed between the two genotypes, as more genes were expressed in the tolerant strain (5,722) than the susceptible strain (4,585); 3,016 shared genes were expressed in both strains (S11 Fig). Further analysis subdivided the shared genes into 22 expression patterns in the WT and WS plants (S12 Fig). The enrichment of GO terms suggested that these genes significantly enhance biological processes related to energy metabolism, such as carbohydrate metabolism, cellular glucan metabolism and oxidation-reduction processes. In addition, such enhanced genes could mediate processes involved in molecular complex generation, including protein polymerization, cellular macromolecular complex assembly, cellulose biosynthesis, and polysaccharide metabolism. These results indicated that energy supply and cell reparation were crucial for sesame recovery following waterlogging stress (S13 Fig). Furthermore, we found enrichment of genes involved in microtubule-based processes and movement, DNA replication and the response to biotic stimuli. Microtubules are critical for a number of cellular processes, as they participate in fundamental aspects of cell division, growth, and differentiation and maintain the structure of the cell by forming the cytoskeleton in concert with microtubule-associated proteins [50]. Microtubules are essential for guard-cell function in *Vicia* and *Arabidopsis* [51]. Guard cells can regulate leaf gas exchange by altering their shape, thereby regulating the stomatal aperture. Thus, microtubules may affect photosynthesis and transpiration in sesame after drainage. The 3,016 shared genes are thought to participate in sesame recovery from waterlogging stress, functioning mainly in energy supply and cell repair.

### Waterlogging response of the homologous stress-responsive and hormone-related genes in sesame

Based on STIFDB V2.0 (Stress Responsive Transcription Factor Database) [28], 1,740 homologous biotic and abiotic stress-responsive genes in sesame compared with *Arabidopsis thaliana* were identified using the RBH method [30, 31]. In total, 968 of these genes were expressed differentially during at least one waterlogging time point, and 1000 DEGs were observed post drainage between WT and WS plants (S14 Fig). Thus, these genes may also function in sesame in response to waterlogging stress. Among these genes, 217 and 187 were detected in the roots of WT and WS, respectively, during waterlogging between 3 and 15 h, with 98 shared genes identified (S5–S7 Tables). Clustering analysis of the 98 shared genes just distinguished the time points under waterlogging (3, 9, and 15 h) from others (0, P20 h) (S15 Fig). The 98 genes were also expected to be included in the 1,379 core waterlogging response genes. This finding suggested the presence of 1,281 newly discovered core responsive genes (S4 Table). Similarly, 276 homologous stress responsive genes were included in the 3,016 genes that expected to participate in recovery from waterlogging stress (S8 Table).

Hormones, such as abscisic acid, auxin, ethylene, gibberellin, salicylic acid, brassinosteroid, cytokinin and jasmonic acid, play essential roles throughout the lifespan of plants and can mediate many pathways required for both biotic resistance and abiotic tolerance [52]. Many genes involved in hormone production and signaling are affected in both the roots and shoots of waterlogged plants. For example, ethylene is involved in the responses to hypoxia and is thought to contribute to adventitious root production and aerenchyma formation [35, 53]. Gibberellin (GA) helps protect *Oryza sativa* L from drowning in deep water by increasing the level of replication protein A1 (RPA1), a heterotrimeric protein involved in the growth of the intercalary meristem of the internode [54]. According to the Arabidopsis Hormone Database 2.0 [29], 601 hormone homologous genes were identified in sesame, and 39 were also members of the aforementioned 1,379 shared DEGs, including abscisic acid, auxin, ethylene, GA, salicylic acid, brassinosteroid, cytokinin and jasmonic acid. Among them, abscisic acid was predominant under waterlogging stress, as 10 of its pathway genes were included (S9 Table). Among the 3,016 genes functioning in recovery, 94 homologous genes representing all eight hormones were detected, and the top three classes included abscisic acid (22), salicylic acid (18), and auxin (16) (S10 Table). These homologous stress-responsive hormone genes may be especially helpful for increasing sesame endurance to waterlogging stress.

### Candidate waterlogging-tolerant genes in sesame

**Fig 4:**
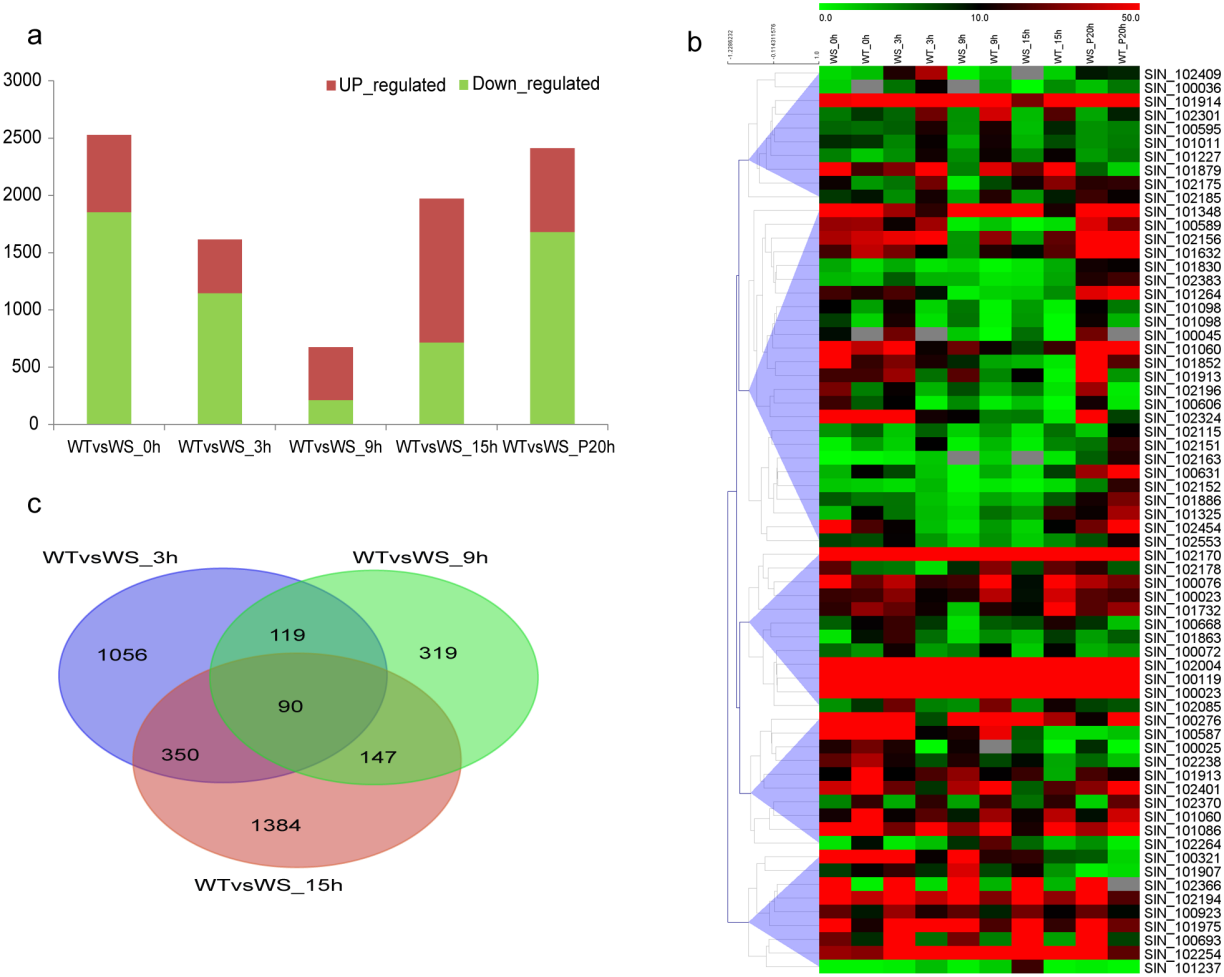
Comparisons between WT and WS plants during waterlogging and post-drainage. (a) DEGs in WT versus WS plants at various waterlogging time points revealed that waterlogging has a severe effect on gene expression at 9 h. (b) Expression patterns of the 66 common DEGs between WT and WS under waterlogging conditions. The RPKM values were log2 transformed. (c) A Venn diagram depicting the common and unique DEGs between 3 and 15 h of waterlogging in WT and WS plants.

The susceptibility of sesame to waterlogging stress is generally attributed to its genotype. At the onset of stress, some sesame genes may begin, increase, or terminate expression to adapt to abnormal nutrient contents in the rhizosphere. In contrast to susceptible sesame, the tolerant genotype may contain genes that are expressed or function differently to improve its survivability in an abiotic environment. In the present study, the number of DEGs between WT and WS plants varied along the time course before and after initiation of waterlogging. Before stress, WT differentially expressed 2,528 genes compared with WS, and 73.3% of the genes were downregulated. After the onset of stress, the number of DEGs decreased until 9 h but consisted mainly of downregulated genes (Fig 4a). Approximately 9 h of waterlogging represented a critical point for sesame, as not only the DEG number between WT and WS decreased to a minimum, but also the number of upregulated genes was more than two-fold the number of downregulated genes. After 9 h of waterlogging, the DEGs between WT and WS increased and tended to equilibrate to a normal status until P20, with the numbers and rates of up- and downregulated genes comparable to those prior to stress. These results reflected that WT responded were more positively than did WS to waterlogging stress.

**Fig 5:**
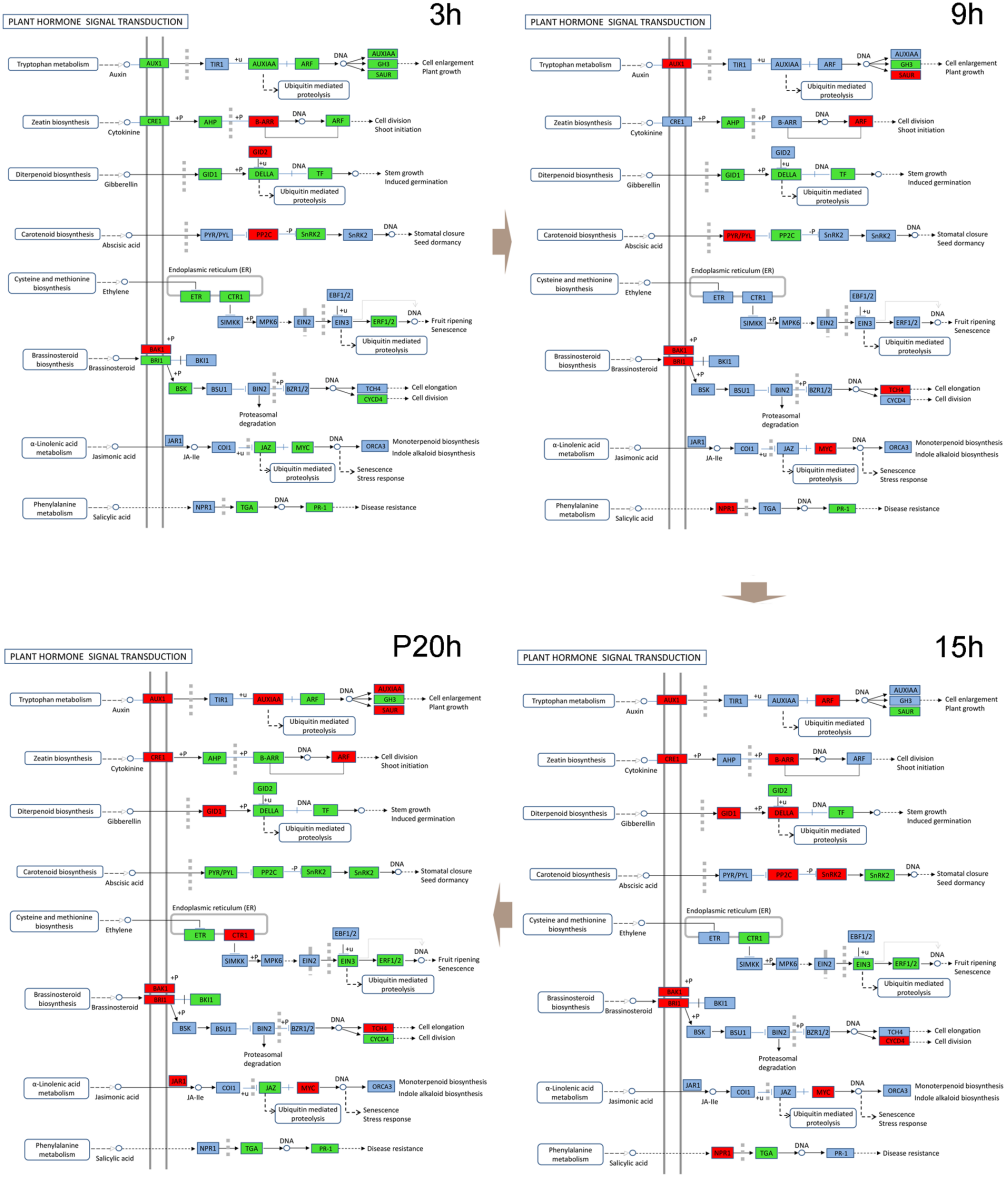
Locations of DEGs between WT and WS in the plant hormone signal transduction pathway. Red indicates the genes upregulated in WT compared with WS plants, and green indicates the downregulated genes.

Annotation for the DEGs between WT and WS at each time point revealed these genes were enriched mainly in pathways involving circadian rhythm, ascorbate and aldarate metabolism, indole alkaloid biosynthesis, flavonoid biosynthesis, phenylpropanoid biosynthesis, plant hormone signal transduction and plant-pathogen interaction. Notably, the enriched categories of phenylpropanoid biosynthesis and plant hormone signal transduction predominated at more than one time point, indicating their particular importance in the WT genotype. The expression profiles of genes in the two pathways indicated that while most genes were expressed at lower levels in WT plants at the beginning of waterlogging, some became more active than those in WS plants with ongoing stress (Figs 5 and S16).

Between 3 and 15 h of waterlogging, 90 DEGs common to WT and WS were detected at all three time points (Fig 4b and 4c). Of these genes, 66 were expressed (RPKM > 10) during at least one time point and may function to improve the endurance of sesame in the face of waterlogging stress. In addition, seven of these genes were differentially expressed prior to waterlogging and 18 expressed post-drainage, suggesting they are long-acting genes involved in guarding against and recovery from waterlogging stress. A hierarchical cluster analysis based on Pearson correlation was used to divide the 66 genes into five groups (Fig 4b), suggesting these genes function in various patterns to enhance the tolerance of the WT genotype. We found five homologous stress-responsive genes (SIN_1024017, SIN_1022545, SIN_1021853, SIN_1021706, and SIN_1012279) among the 66 genes, and their expression patterns were validated by RT-PCR (S17 Fig). Notably, SIN_1021706 encodes a gene homologous to a hormone with abscisic acid 8'-hydroxylase activity involved in abscisic acid catabolism. Mutant analyses demonstrated that disruption of this gene results in increased drought tolerance [55, 56]. The expression of SIN_1021706 was increased in WT plants but downregulated in WS plants during waterlogging. In addition, the 66 active genes were highlighted in the functional categories of chitin catabolic processing, cell wall macromolecule catabolic processing with chitin binding, chitinase activity, and carbohydrate binding (S11 Table). Chitin is a characteristic component that contributes significantly to the mechanical strength of the cell wall, and chitin synthesis is essential for the plant’s response to environmental stress [57–59]. Therefore, these genes are associated with strengthening the endurance of sesame to waterlogging stress. Therefore, these genes are candidate waterlogging-tolerance genes that should be studied further.

## Conclusions

Sesame is affected easily and negatively by waterlogging. The present study provides comprehensive insight into the genetic response of sesame to waterlogging stress over time. Waterlogging stress gradually suppresses the expression of sesame genes, with approximately half being significantly induced or suppressed. We found that ~9 h after the onset of waterlogging stress was a critical time point for sesame, providing useful information for farmers in planning how to ameliorate or prevent sesame water damage. The fact that more genes in sesame than in other species were affected by waterlogging demonstrated the high susceptibility of sesame. However, sesame of different genotypes exhibited differential response patterns under waterlogging stress; the tolerant species remained more stable throughout the waterlogging. The core gene set involved in the sesame response to waterlogging stress—and in particular, the newly discovered responsive genes—will be useful in expanding our knowledge of the mechanism underlying waterlogging stress in sesame. These data also revealed that energy metabolism and phenylpropanoid biosynthesis play crucial roles in regulating sesame endurance against waterlogging stress. Based on the comparison between tolerant and susceptible genotypes, a list of candidate genes for improving sesame tolerance to waterlogging stress was generated, which will serve as a guideline for future research. However, these genes require further verification in additional experiments. Future studies utilizing independent approaches—such as transcriptomics, proteomics, physiology, biochemistry, metabolite profiling, and genetic analysis—will lead to a better understanding of the response of sesame to waterlogging stress and will aid the effort to develop waterlogging-tolerant sesame.

## Supporting Information

S1 Fig Clean data for 10 sesame transcriptomes acquired by next generation sequencing technology. (TIF)

S2 Fig Distributions of gene coverage. (TIF)

S3 Fig Distributions of reads among gene regions. (TIF)

S4 Fig Venn diagram depicting the genes commonly expressed in WT and WS plants at each time point. (TIF)

S5 Fig GO-SLIM categories for the genes commonly expressed in both WT and WS plants. BP: biological processes; MF: molecular function; CC: cellular component.
(TIF)

S6 Fig Statistics of the expressed genes in each sample during waterlogging and post-drainage. (TIF)

S7 Fig GO-SLIM categories of the insensitive genes commonly expressed in both WT and WS plants. BP: biological processes; MF: molecular function; CC: cellular component.
(TIF)

S8 Fig Expression patterns of the 18 clusters of the 1,379 core genes responsive to waterlogging. (TIF)

S9 Fig The positions of the core responsive genes in the carbon fixation pathway in photosynthetic organisms. (TIF)

S10 Fig The positions of the core responsive genes in the glycolysis and gluconeogenesis pathway. (TIF)

S11 Fig Venn diagrams of the unique and common DEGs in the WT and WS genotypes at 20 h post-waterlogging. (TIF)

S12 Fig Expression patterns of the 3,016 core genes responsible for recovery from waterlogging in sesame. (TIF)

S13 Fig GO categories of the enriched core gene set involved in recovery after drainage. BP: biological processes; MF: molecular function; CC: cellular component.
(TIF)

S14 Fig Expression profiles of sesame homologous stress-responsive genes during and post-waterlogging. (TIF)

S15 Fig Expression patterns of the 98 homologous stress-responsive genes. (TIF)

S16 Fig DEGs between WT and WS plants in the phenylpropanoid biosynthesis pathway. (TIF)

S17 Fig RT-PCR validation of the genes in WT and WS. (TIF)

S1 Table The samples and their appointed names used in the present study. (XLSX)

S2 Table Overview of the data generated. (XLSX)

S3 Table GO enrichment of the 5,397 genes insensitive to waterlogging. (XLSX)

S4 Table The 1,379 core genes involved in sesame response to waterlogging. (XLSX)

S5 Table 217 homologous stress responsive genes differently expressed in WT plants during waterlogging from 3 to 15 h. (XLSX)

S6 Table 187 homologous stress-responsive genes differentially expressed in WS during waterlogging between 3 and 15 h. (XLSX)

S7 Table 98 shared homologous stress-responsive DEGs in both WT and WS plants during waterlogging between 3 and 15 h. (XLSX)

S8 Table 276 homologous stress-responsive genes differentially expressed in both WT and WS plants post-drainage. (XLSX)

S9 Table List of the 39 core hormone homologous genes responsive to waterlogging in sesame. (XLSX)

S10 Table List of the 94 hormone homologous genes functioning post-drainage in sesame. (XLSX)

S11 Table GO enrichment of the 66 common DEGs between WT and WS under waterlogging from 3 to 15h. (XLSX)

## Abbreviations

DEG: differentially expressed gene
FDR: false discovery rate
GO: gene ontology
IGPD: imidazoleglycerol-phosphate dehydratase
KEGG: kyoto encyclopedia of genes and genomes
RPKM: reads per kilo base of exon model per million mapped reads
WT: waterlogging-tolerant sesame
WS: waterlogging-susceptible sesame
