# Supplementary figures and images for: Tolerant and Susceptible Sesame Genotypes Reveal Waterlogging Stress Response Patterns
Source: PLoS One. 2016 Mar 2;11(3):e0149912. doi: 10.1371/journal.pone.0149912 (PMC4774966; doi:10.1371/journal.pone.0149912)

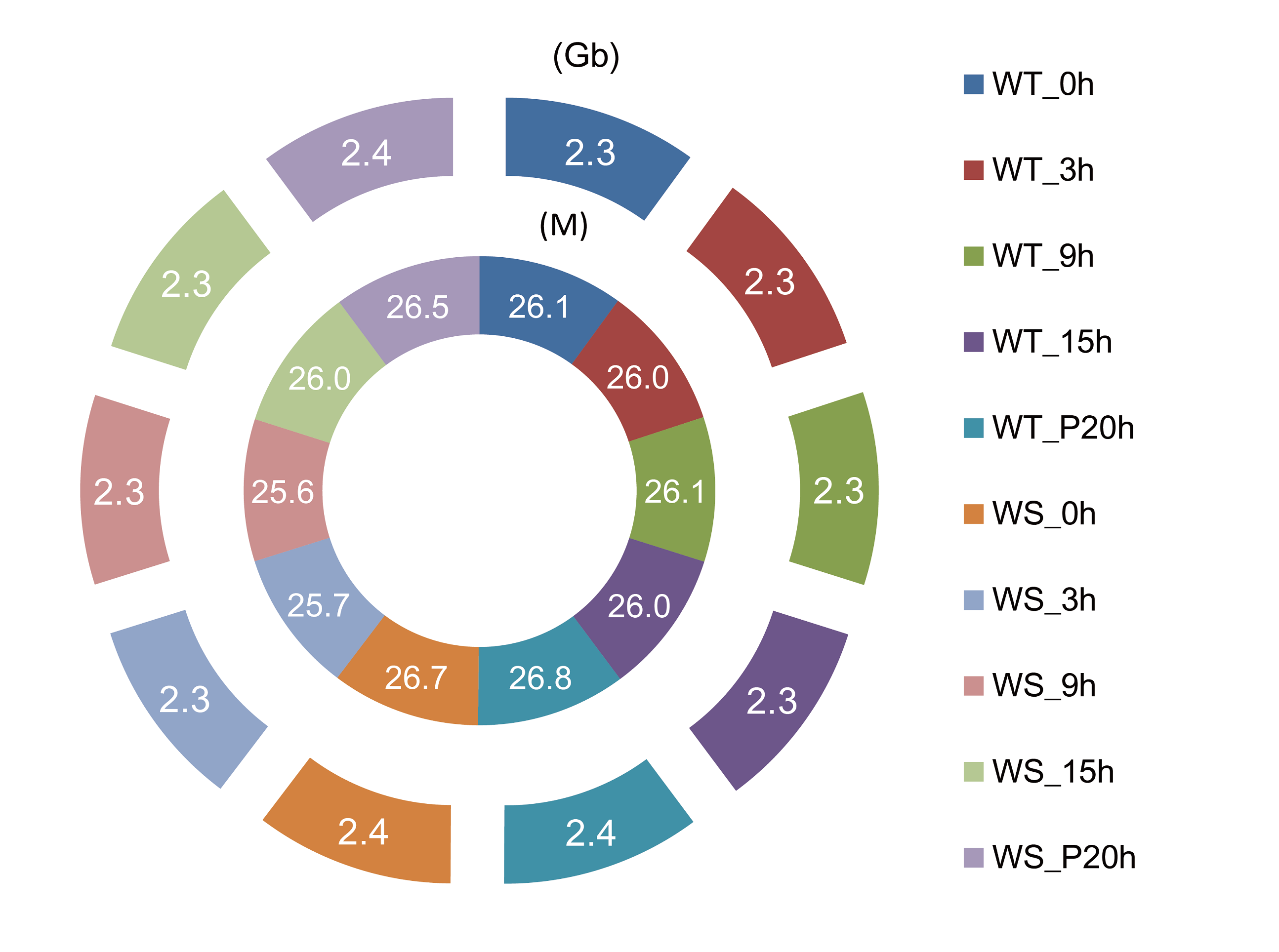

Supplement: S1 Fig — (TIF) [file pone.0149912.s001.tif]

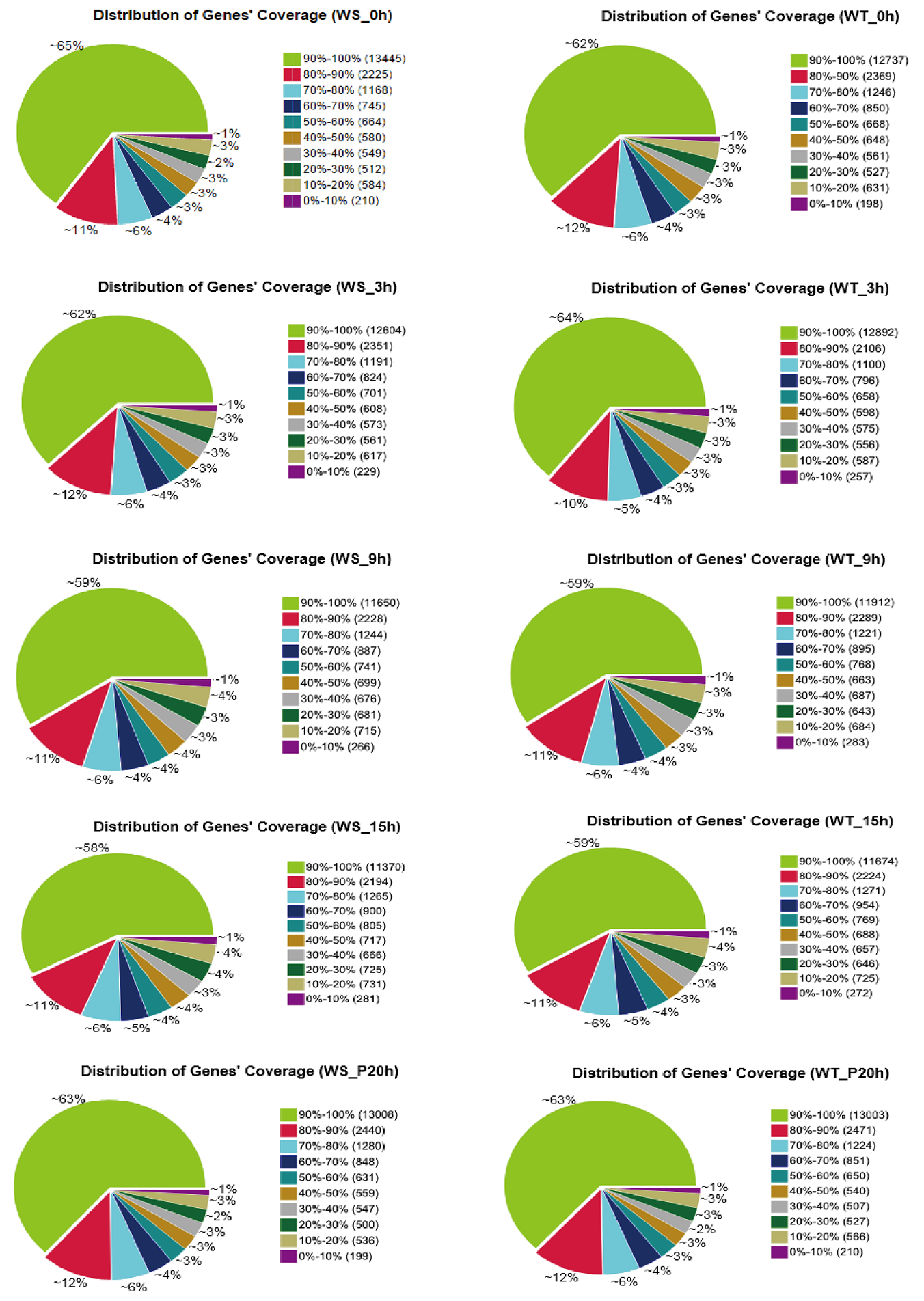

Supplement: S2 Fig — (TIF) [file pone.0149912.s002.tif]

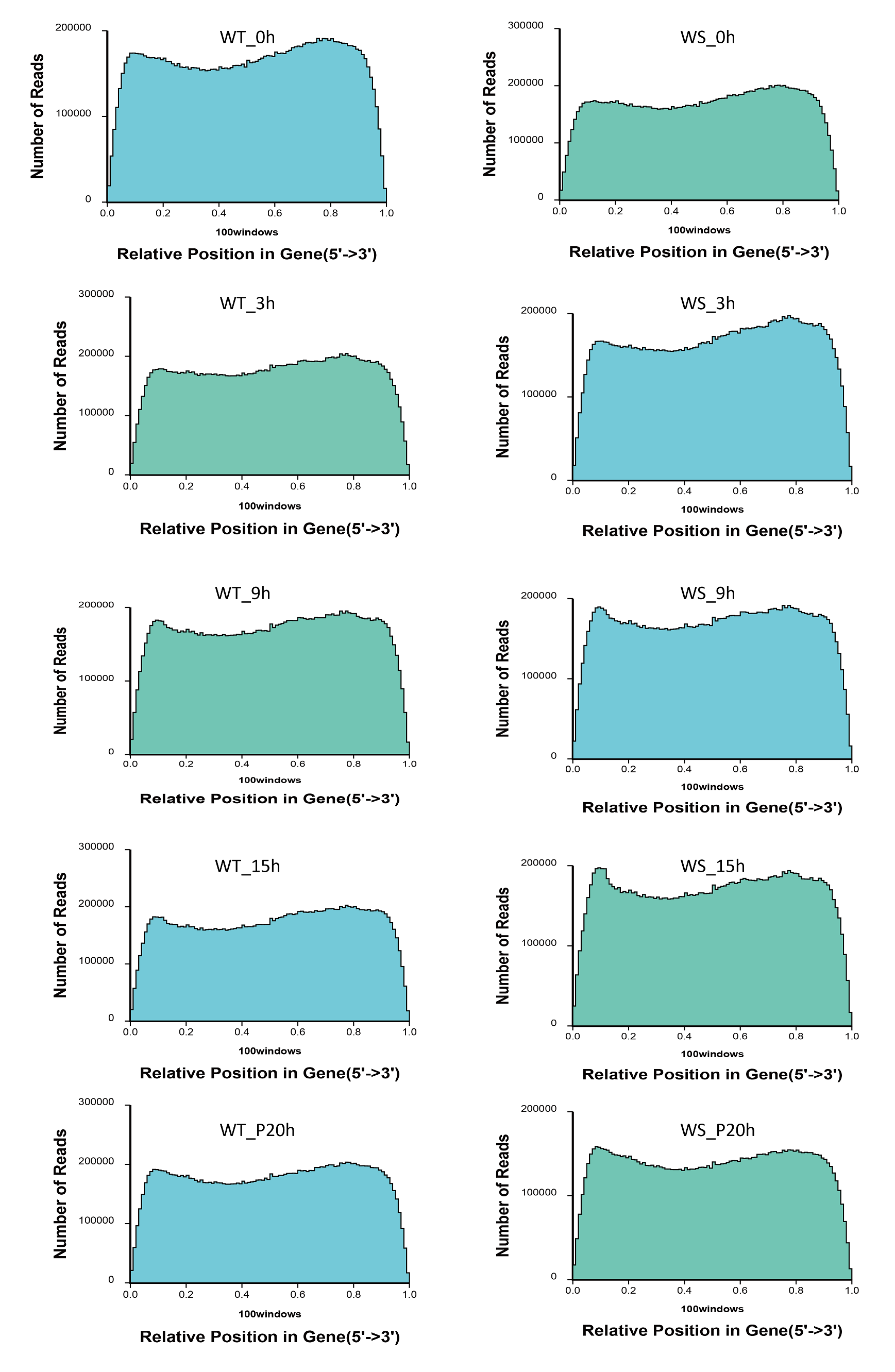

Supplement: S3 Fig — (TIF) [file pone.0149912.s003.tif]

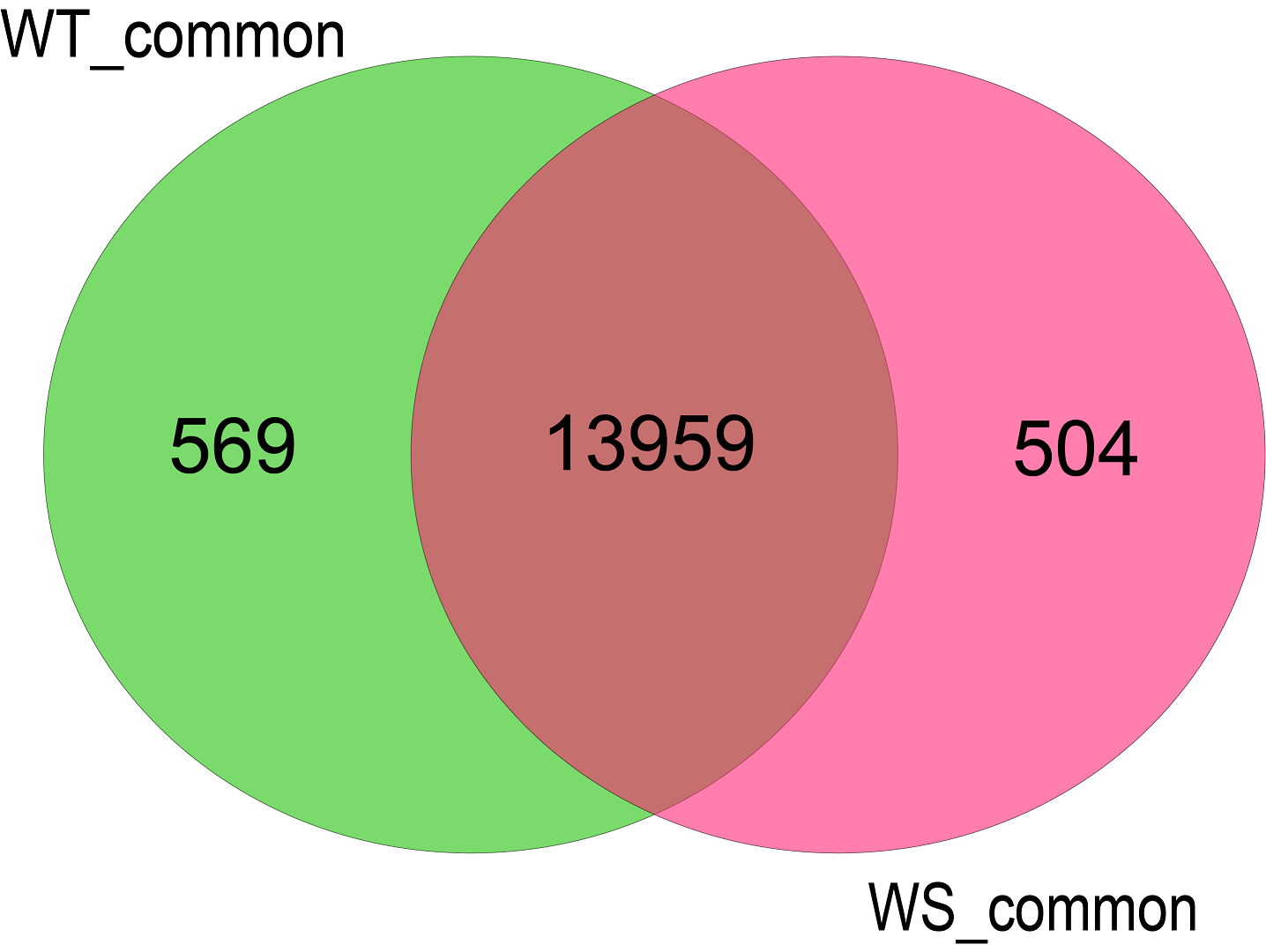

Supplement: S4 Fig — (TIF) [file pone.0149912.s004.tif]

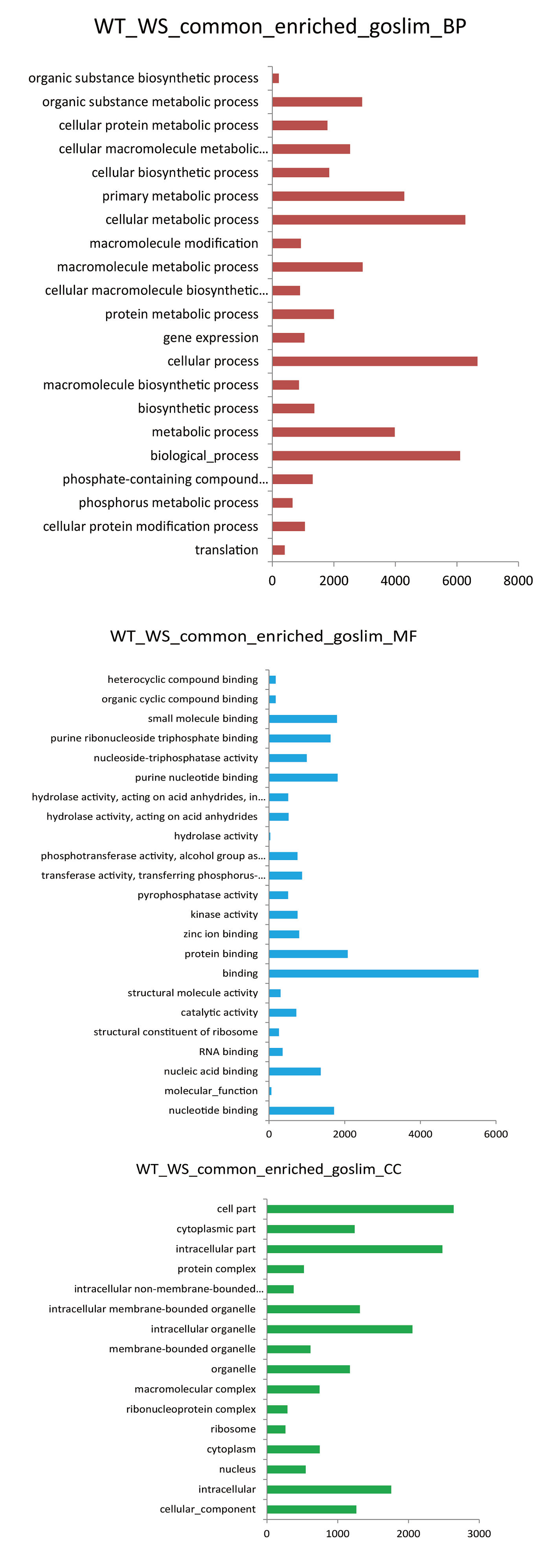

Supplement: S5 Fig — BP: biological processes; MF: molecular function; CC: cellular component. (TIF) [file pone.0149912.s005.tif]

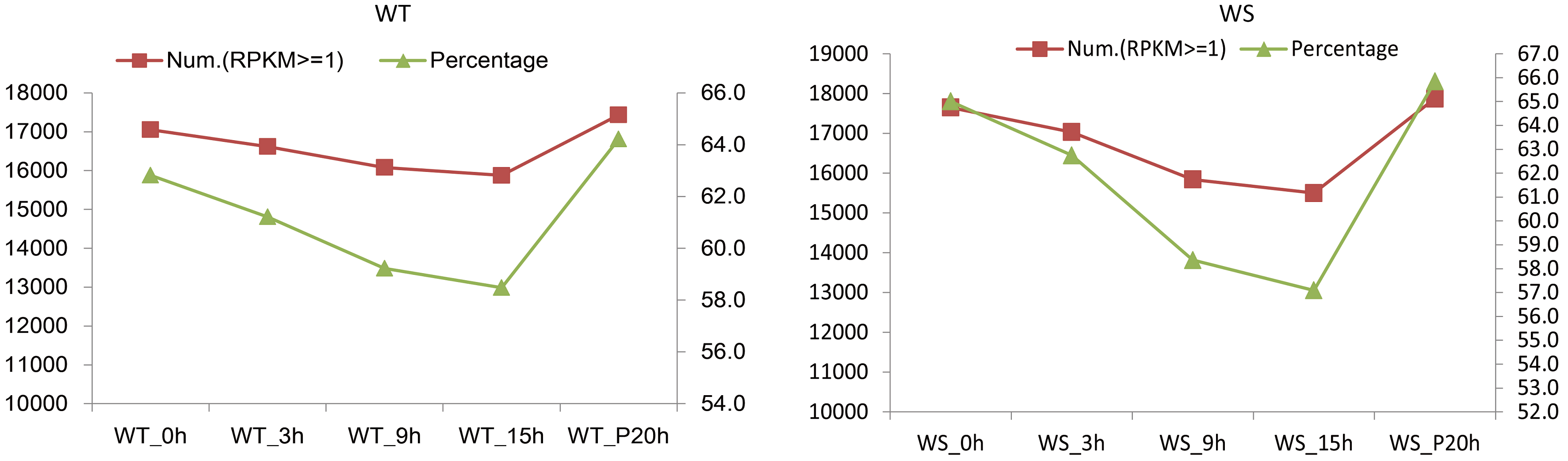

Supplement: S6 Fig — (TIF) [file pone.0149912.s006.tif]

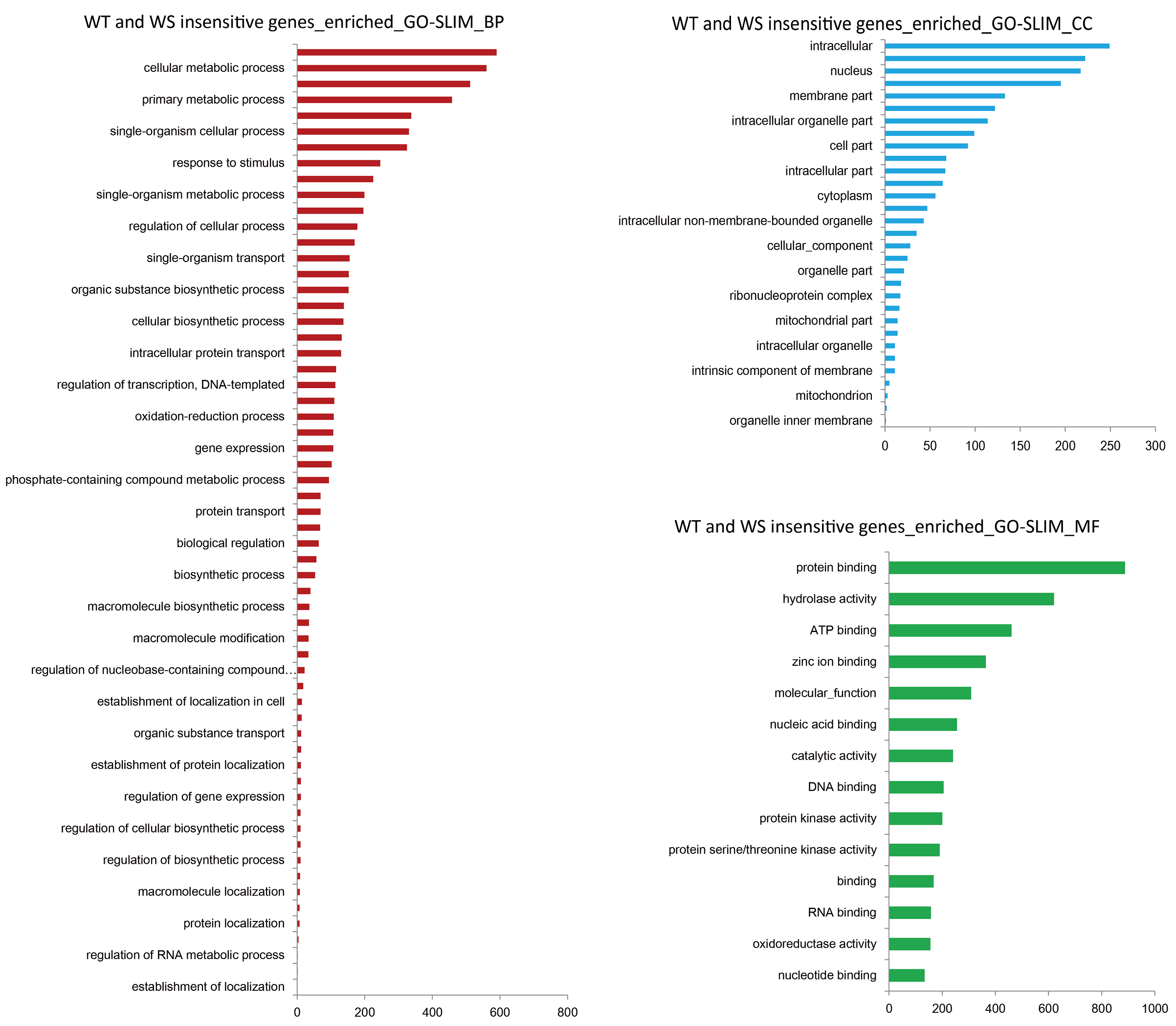

Supplement: S7 Fig — BP: biological processes; MF: molecular function; CC: cellular component. (TIF) [file pone.0149912.s007.tif]

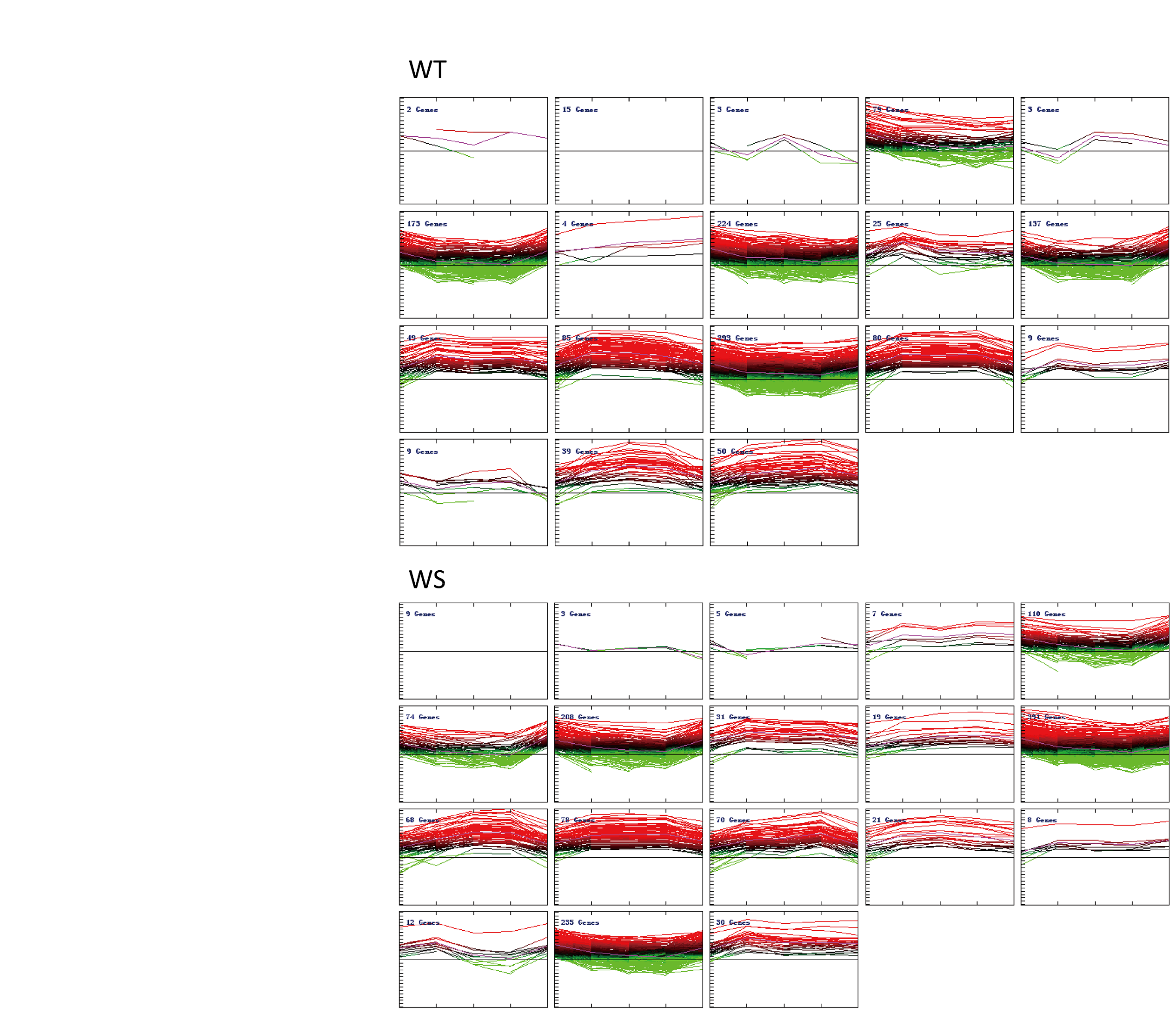

Supplement: S8 Fig — (TIF) [file pone.0149912.s008.tif]

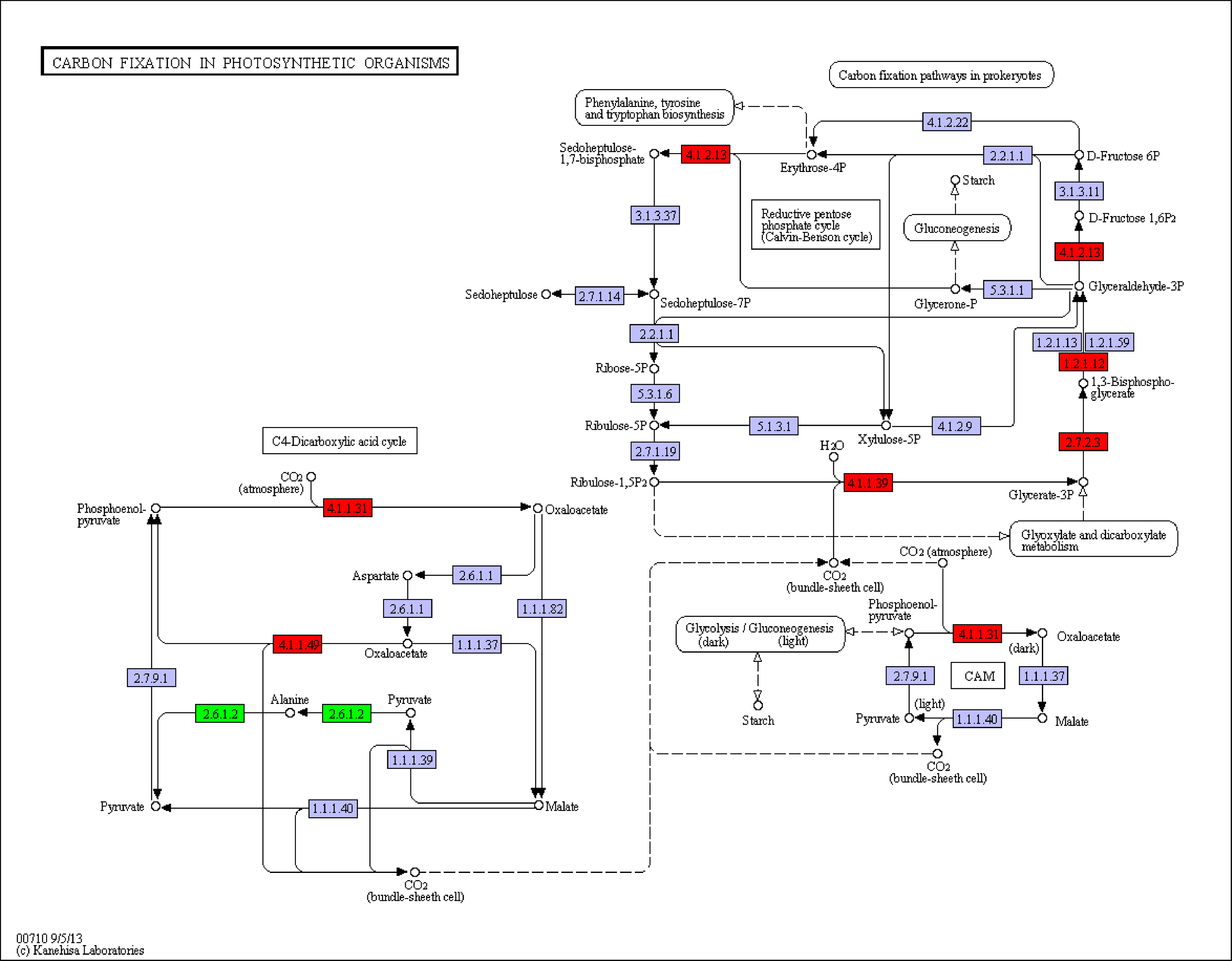

Supplement: S9 Fig — (TIF) [file pone.0149912.s009.tif]

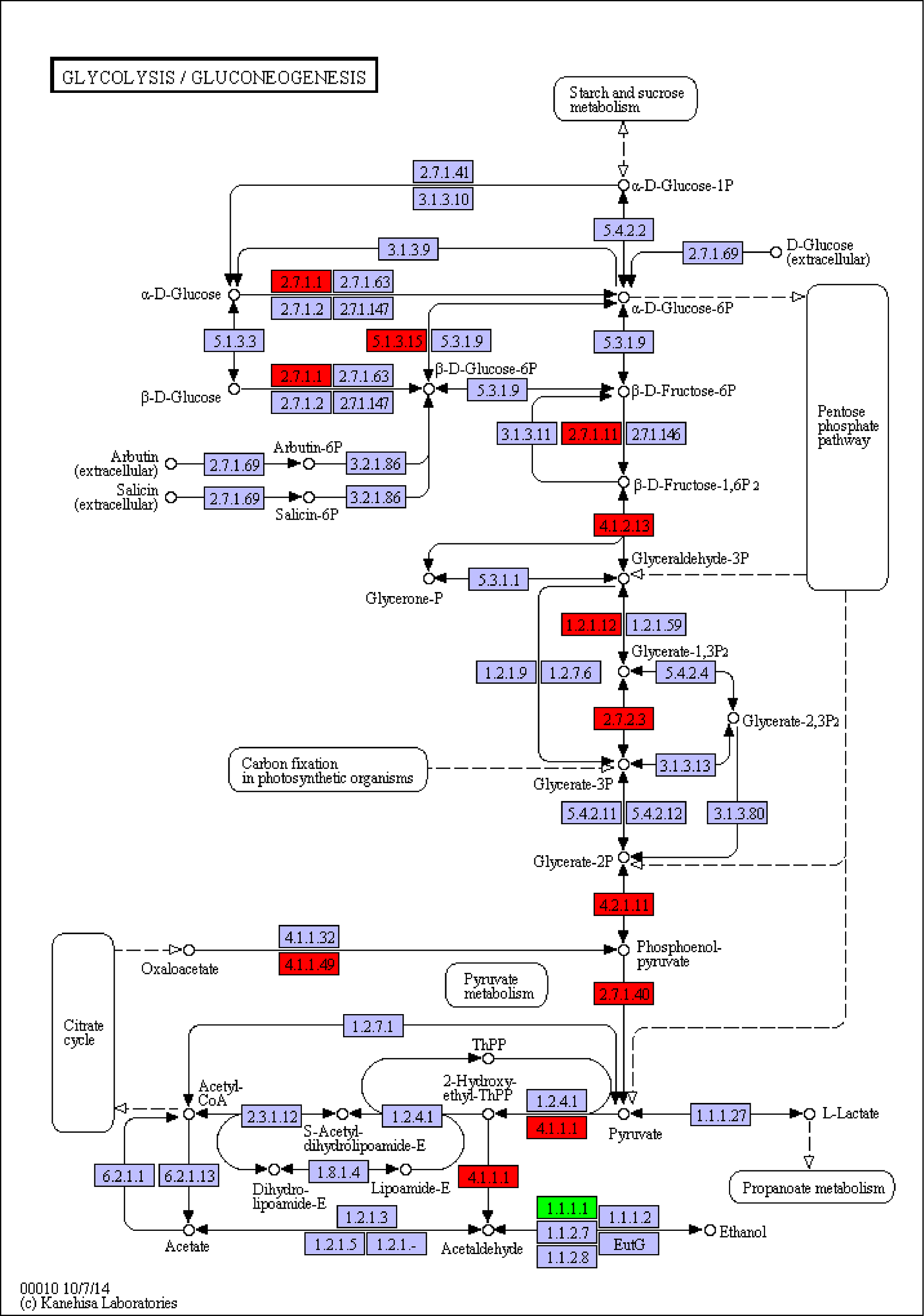

Supplement: S10 Fig — (TIF) [file pone.0149912.s010.tif]

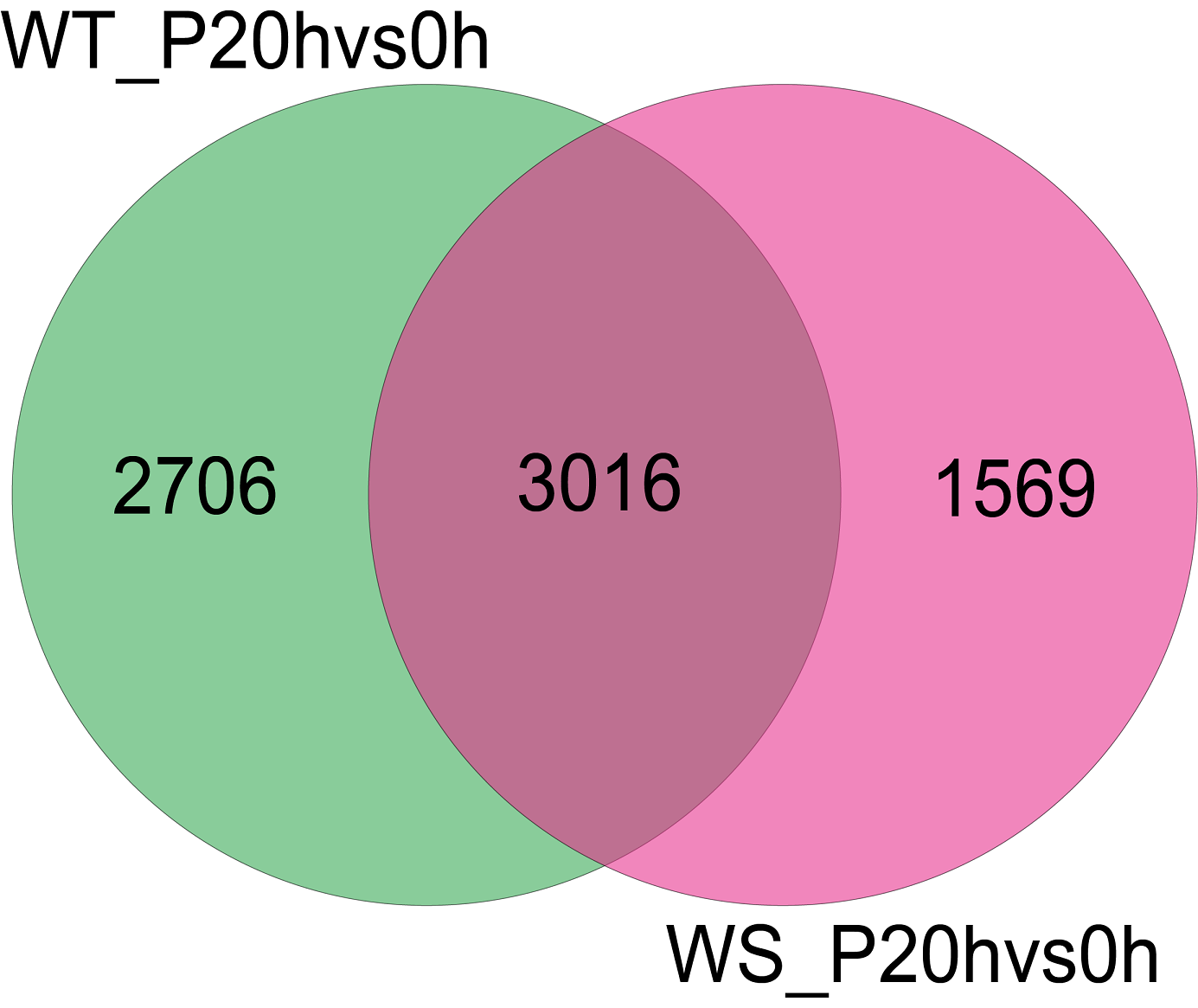

Supplement: S11 Fig — (TIF) [file pone.0149912.s011.tif]

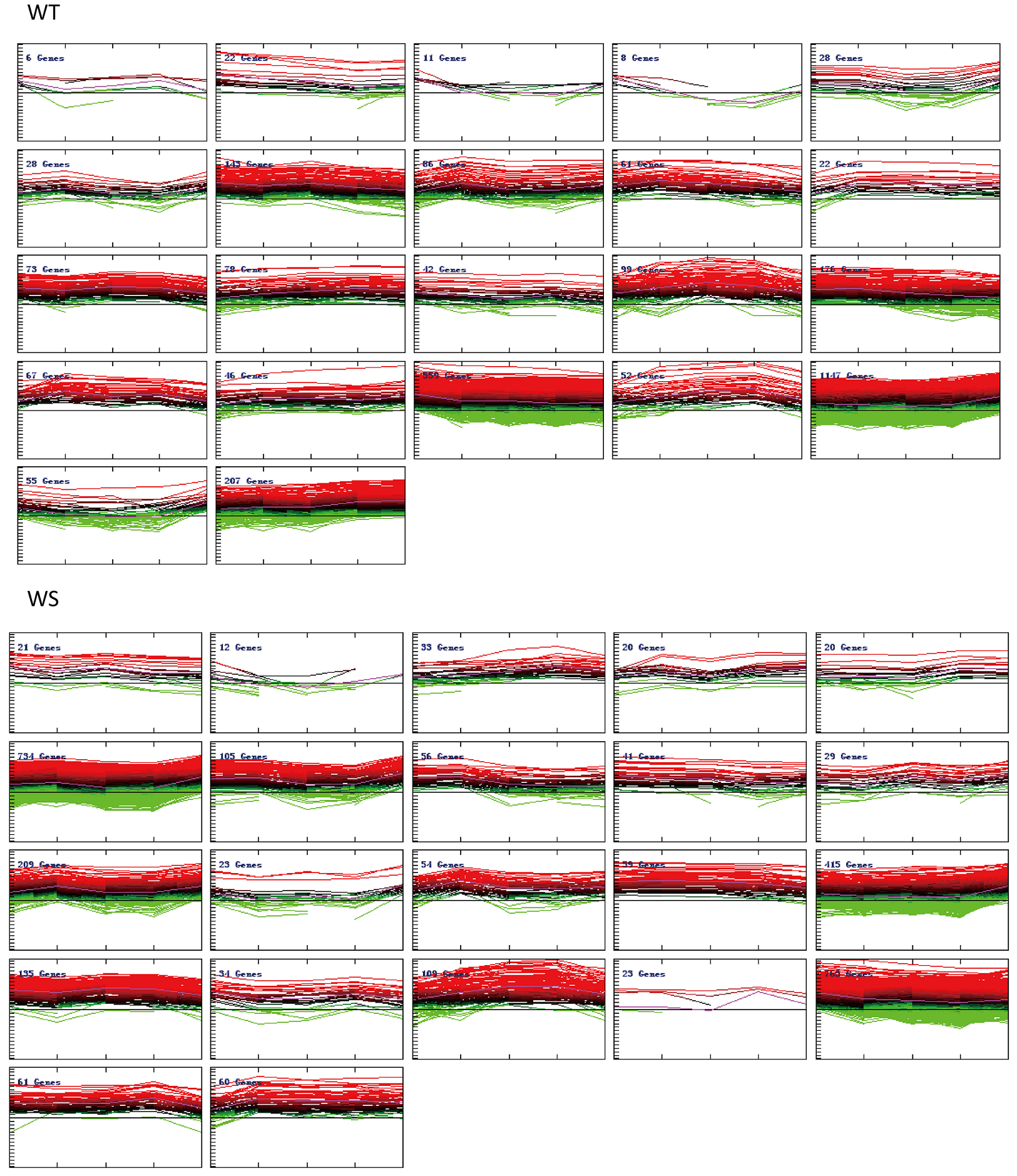

Supplement: S12 Fig — (TIF) [file pone.0149912.s012.tif]

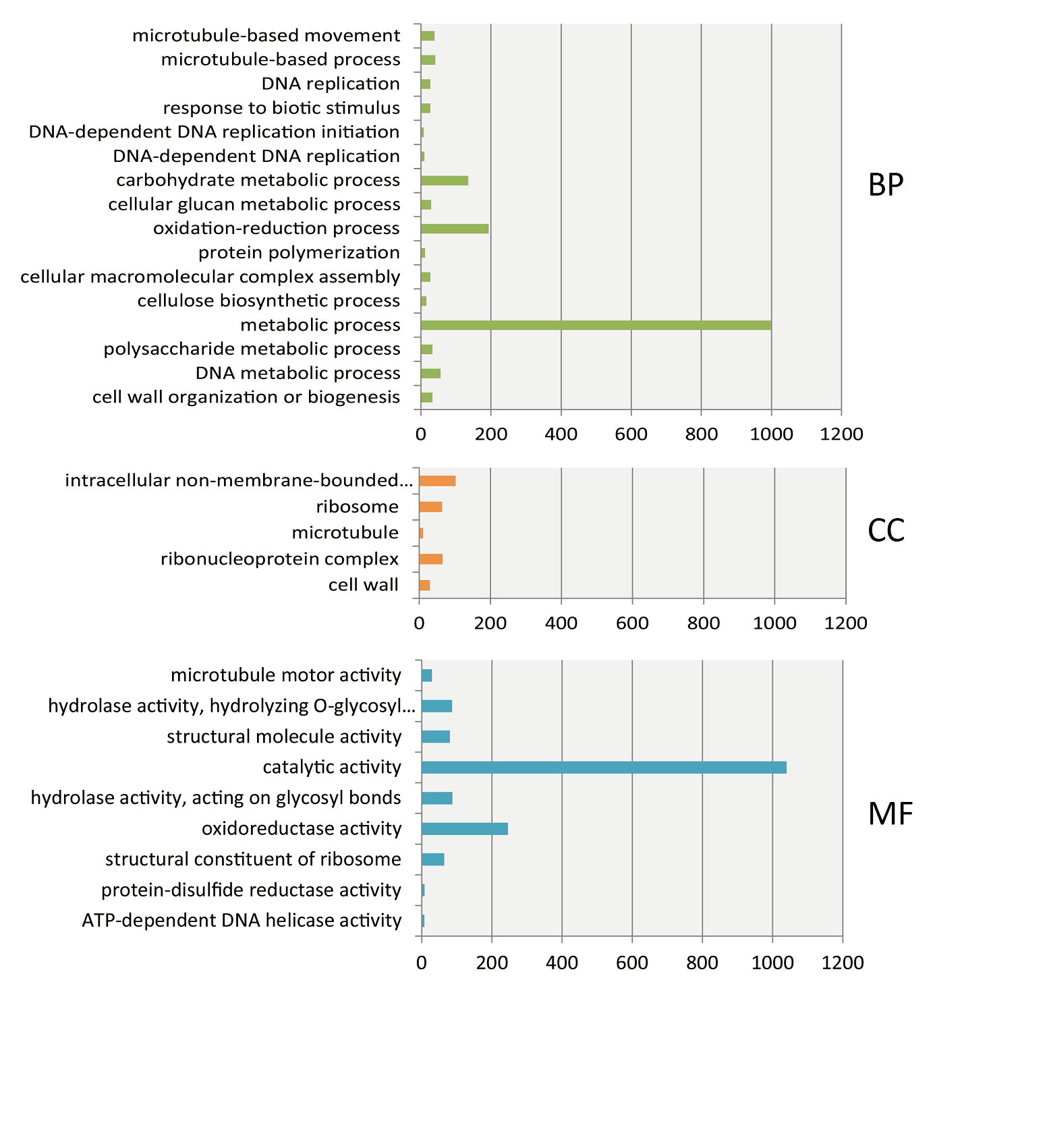

Supplement: S13 Fig — BP: biological processes; MF: molecular function; CC: cellular component. (TIF) [file pone.0149912.s013.tif]

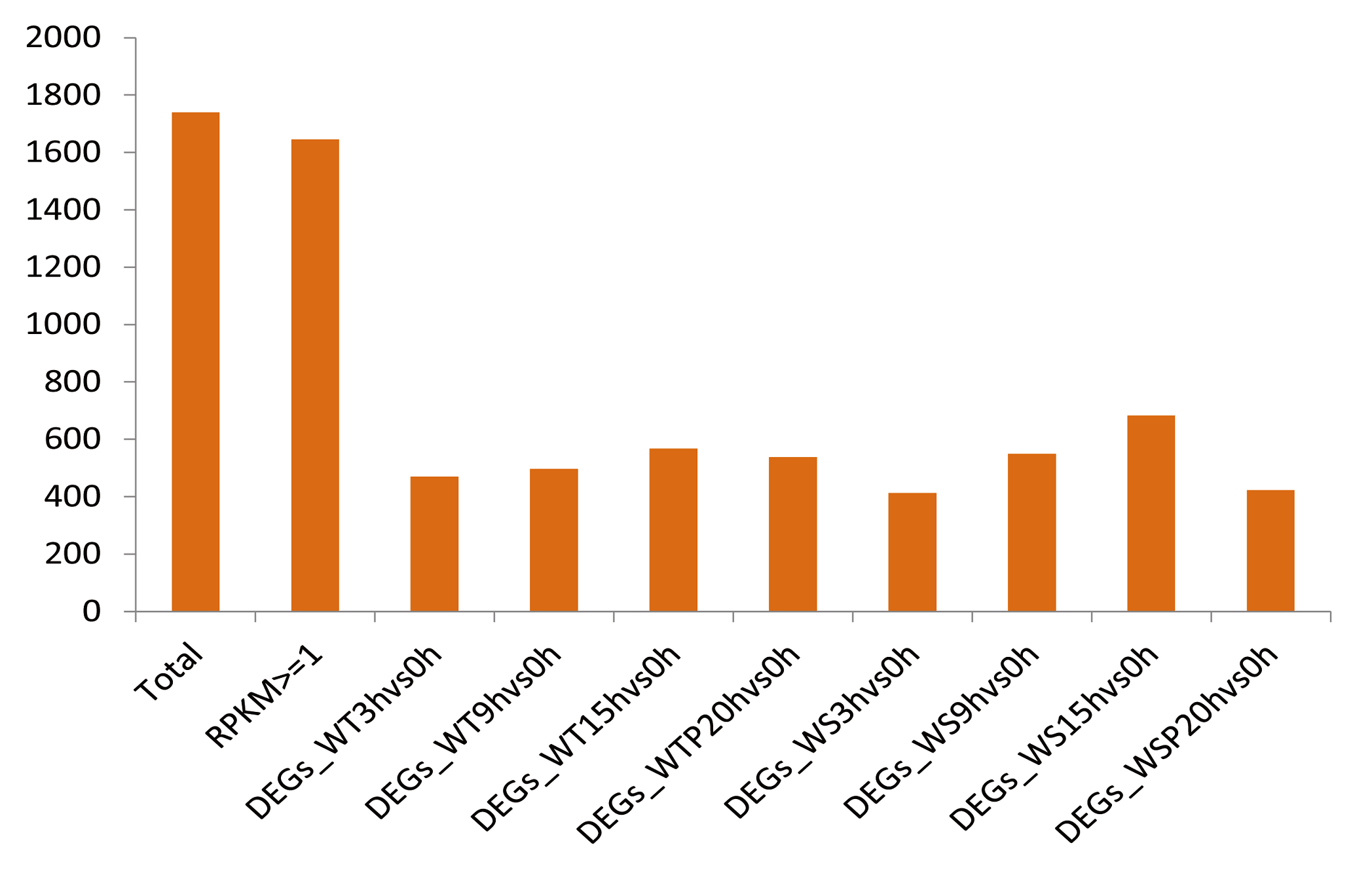

Supplement: S14 Fig — (TIF) [file pone.0149912.s014.tif]

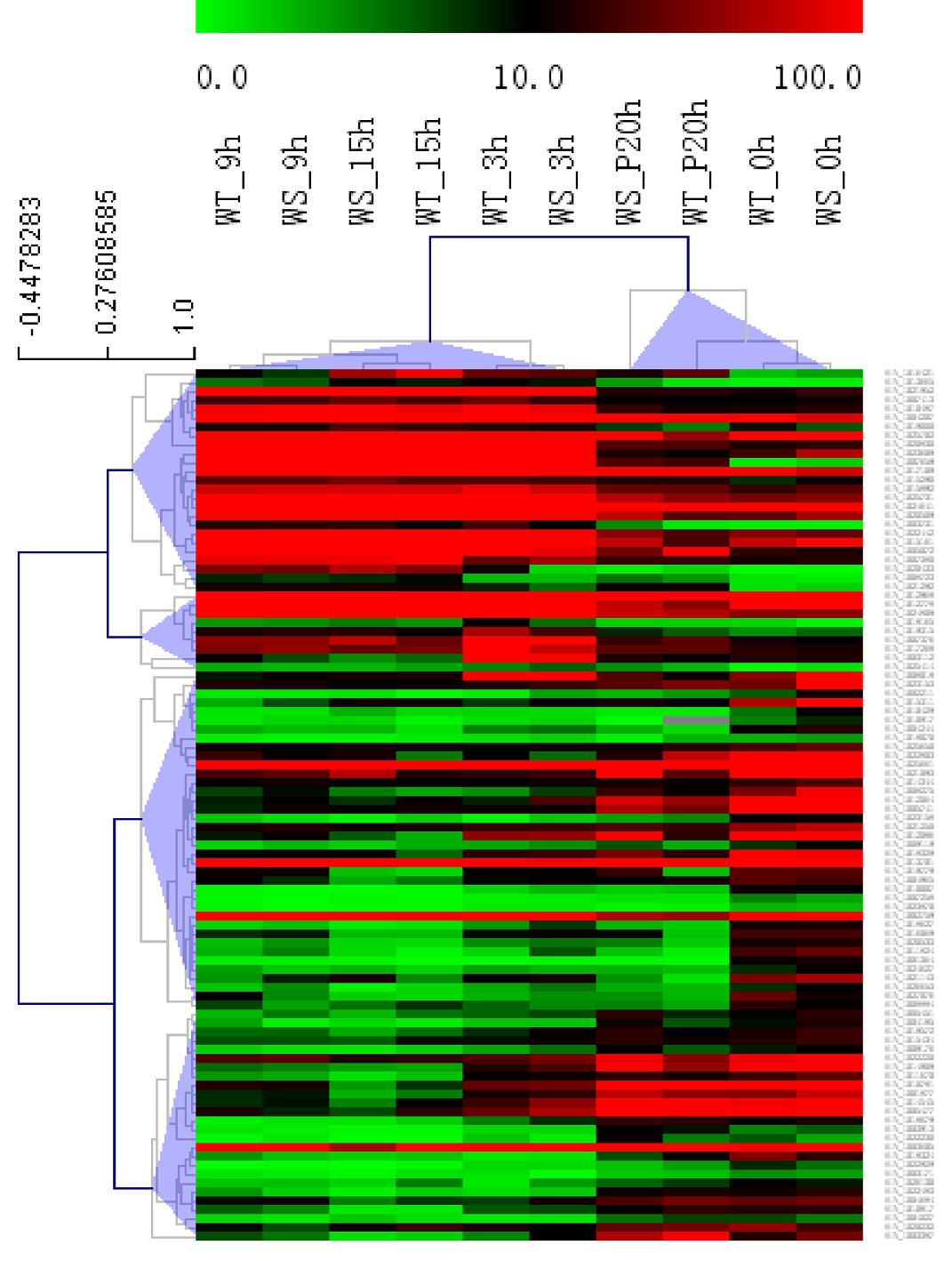

Supplement: S15 Fig — (TIF) [file pone.0149912.s015.tif]

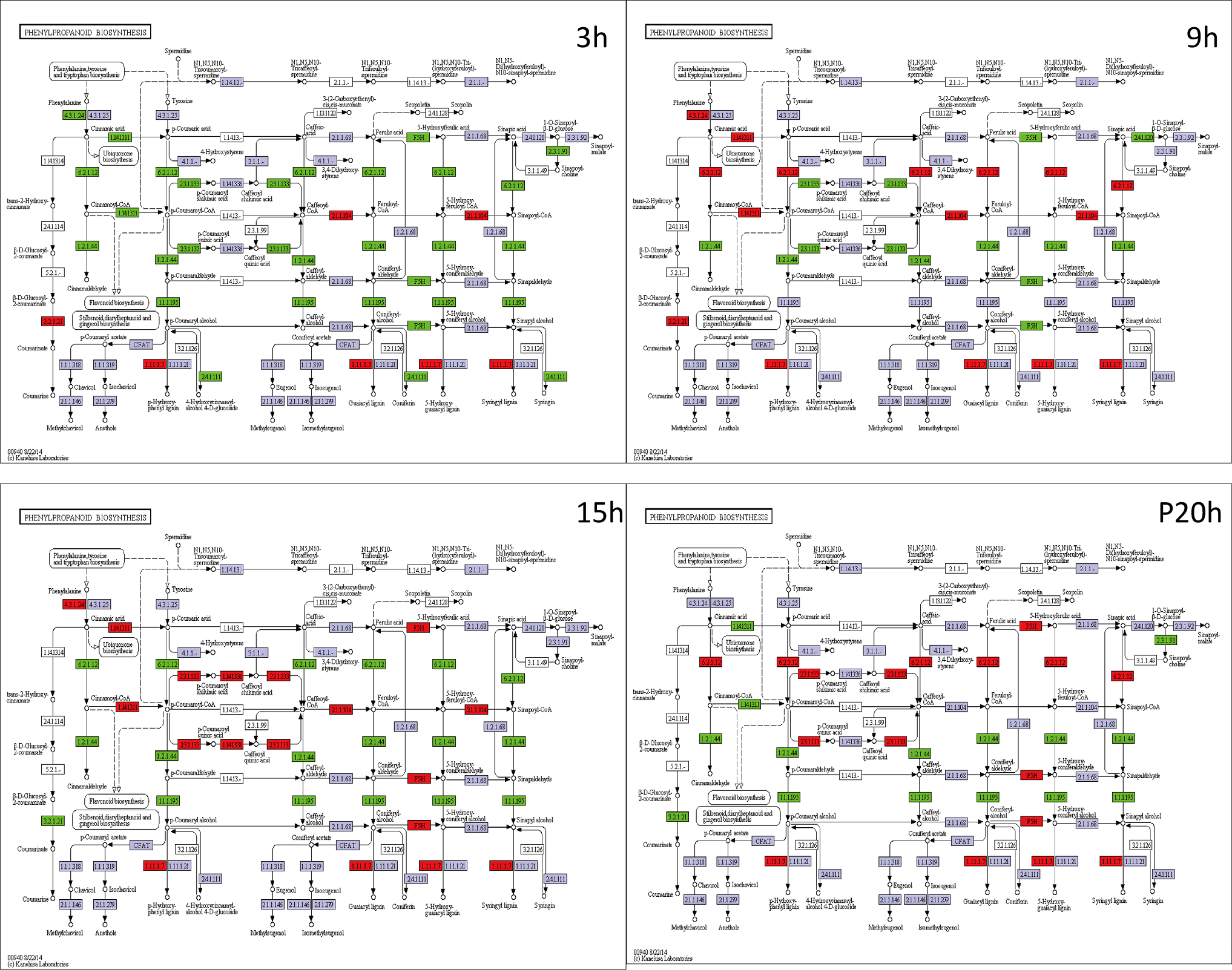

Supplement: S16 Fig — (TIF) [file pone.0149912.s016.tif]

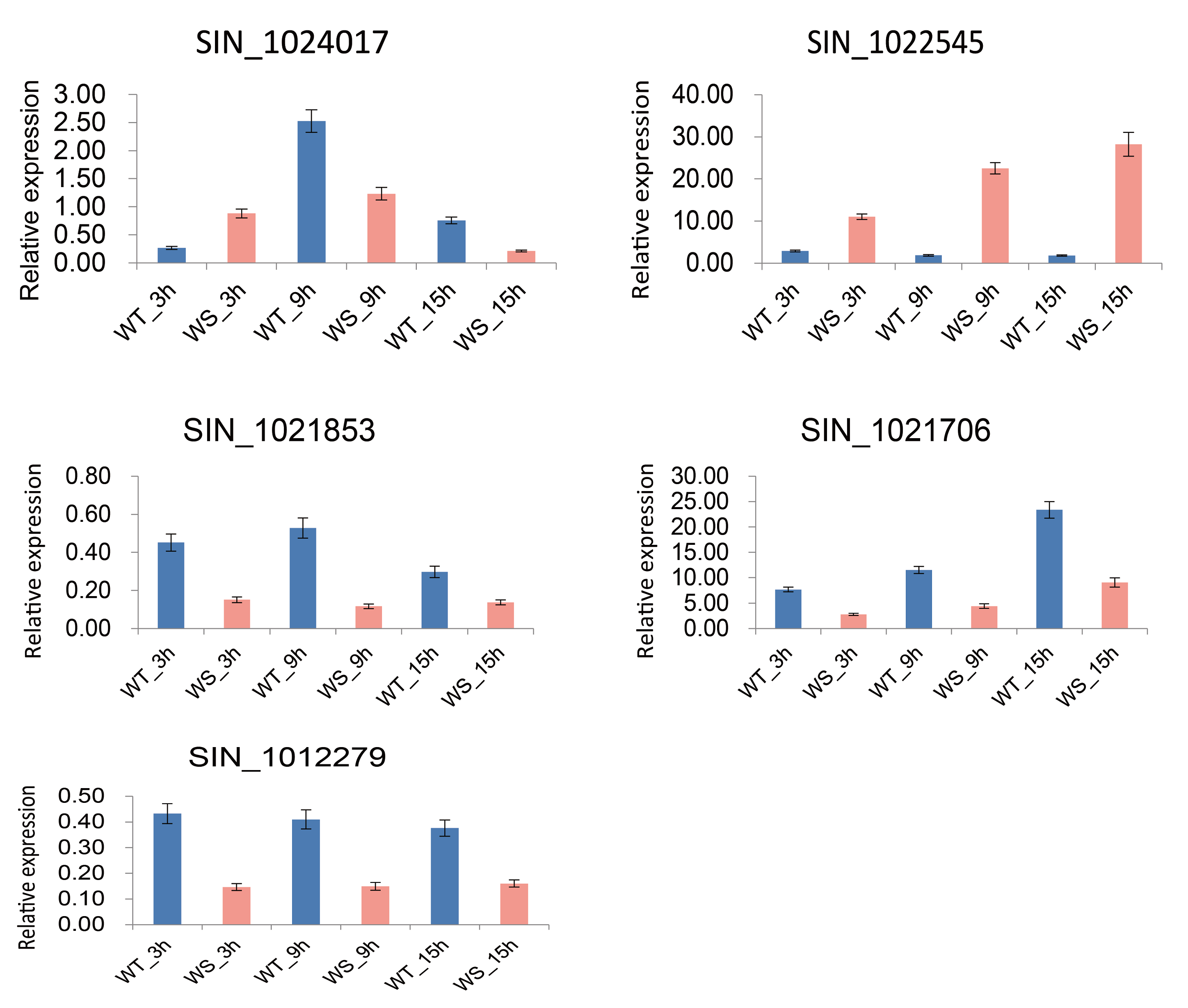

Supplement: S17 Fig — (TIF) [file pone.0149912.s017.tif]
